# Electronic Tagging of Atlantic Bluefin Tuna (*Thunnus thynnus*, L.) Reveals Habitat Use and Behaviors in the Mediterranean Sea

**DOI:** 10.1371/journal.pone.0116638

**Published:** 2015-02-11

**Authors:** Pablo Cermeño, Gemma Quílez-Badia, Andrés Ospina-Alvarez, Susana Sainz-Trápaga, Andre M. Boustany, Andy C. Seitz, Sergi Tudela, Barbara A. Block

**Affiliations:** 1 Department of Biology, Stanford University, Hopkins Marine Station, Pacific Grove, California 93950, United States of America; 2 WWF Mediterranean Programme, Barcelona 08002, Spain; 3 Center for Marine Conservation & Department of Ecology, Biological sciences Faculty, Pontificia Universidad Católica de Chile, Santiago de Chile 6513677, Chile; 4 Nicholas School of the Environment, Duke University, Durham, North Carolina 27708, United States of America; 5 Tuna Research and Conservation Center, Monterey Bay Aquarium, Monterey, California 93940, United States of America; 6 School of Fisheries and Ocean Sciences, University of Alaska Fairbanks, Fairbanks, Alaska 99775, United States of America; Institut Pluridisciplinaire Hubert Curien, FRANCE

## Abstract

We analyzed the movements of Atlantic tuna (*Thunnus thynnus* L.) in the Mediterranean Sea using data from 2 archival tags and 37 pop-up satellite archival tags (PAT). Bluefin tuna ranging in size from 12 to 248 kg were tagged on board recreational boats in the western Mediterranean and the Adriatic Sea between May and September during two different periods (2000 to 2001 and 2008 to 2012). Although tuna migrations between the Mediterranean Sea and the Atlantic Ocean have been well reported, our results indicate that part of the bluefin tuna population remains in the Mediterranean basin for much of the year, revealing a more complex population structure. In this study we demonstrate links between the western Mediterranean, the Adriatic and the Gulf of Sidra (Libya) using over 4336 recorded days of location and behavior data from tagged bluefin tuna with a maximum track length of 394 days. We described the oceanographic preferences and horizontal behaviors during the spawning season for 4 adult bluefin tuna. We also analyzed the time series data that reveals the vertical behavior of one pop-up satellite tag recovered, which was attached to a 43.9 kg tuna. This fish displayed a unique diving pattern within 16 days of the spawning season, suggesting a use of the thermocline as a thermoregulatory mechanism compatible with spawning. The results obtained hereby confirm that the Mediterranean is clearly an important habitat for this species, not only as spawning ground, but also as an overwintering foraging ground.

## Introduction

Atlantic bluefin tuna are the largest members of the family Scombridae, with adult tuna reaching lengths of 3.31 m and a mass up to 725 kg [1]. The Atlantic bluefin tuna (*Thunnus thynnus*) has been a focus of marine fisheries research and a target of fishers since ancient times [2,3]. While sustainable fisheries for bluefin tuna have existed in the Mediterranean Sea for centuries, fishing mortality rates of this large predator escalated in recent decades [4]. This resulted in a sharp decline of the populations in the past four decades [5] and the risk of collapse of breeding populations both within the Gulf of Mexico and the Mediterranean Sea [6,7]. Improving fisheries models of Atlantic bluefin tuna population structure, migration patterns and habitat use is necessary, particularly on their spawning grounds where they are heavily exploited. Understanding habitat utilization is critical for the design of more effective management measures.

The Atlantic bluefin tuna have two known spawning grounds where reproductively active adults and larvae have been consistently found, i.e. the Gulf of Mexico/Caribbean/Straits of Florida (hereafter referred to as Gulf of Mexico) and the Mediterranean Sea [8]. Spawning may occur in other regions in the western Atlantic but to date, larval specimens are few and are recovered adjacent to known spawning regions [9]. The species has been managed by the International Commission for the Conservation of Atlantic Tuna (ICCAT) essentially as two populations, the western and the eastern populations with their respective breeding areas (Gulf of Mexico and the Mediterranean Sea), separated by the 45°W meridian [4]. Electronic tagging, otolith and genetic markers have shown that there is an extensive degree of mixing between distinct populations on the North Atlantic foraging grounds [10–14]. Genetic studies suggest there are at least three distinct populations throughout its geographical range [15–17] that most likely represents Gulf of Mexico, western Mediterranean and eastern Mediterranean populations.

Mature bluefin tuna of eastern origin undertake two types of migrations: movements into the Mediterranean Sea to spawn, which occurs during April and May [18], and a post-spawning foraging migration to the North Atlantic Ocean, around the end of July and August [19–21]. Studies based on electronic archival and pop-up satellite archival tags in the past decade have been instrumental in identifying the details of bluefin tuna movement patterns, vertical distributions, temperature preferences and feeding habits. Most of these studies, however, have been carried out in the western Atlantic Ocean [10–13,16, 22–29] with over 2000 electronic tags having been deployed since the late 1990s. Interestingly, a significant number of Atlantic bluefin tuna archivally tagged in the western Atlantic, particularly in the coastal waters off North Carolina (eastern North America), displayed site directed fidelity to the western Mediterranean spawning grounds. This has been hypothesized to indicate eastern origin fish make trans-Atlantic migrations as juveniles but return to Mediterranean oceanic regions for spawning [13]. Only once has an Atlantic bluefin tuna tagged in the western Atlantic had a verified recapture to the east of the Adriatic Sea [12,13,26]. These fish presumably are spawned in the Mediterranean Sea, occupy the West Atlantic Ocean as juveniles presumably foraging on large schools of herring and menhaden, and then move back into the Mediterranean as they mature into spawners. Once spawning occurs electronic tagging has shown site directed fidelity to the western Mediterranean and western Atlantic [13].

Most of the Atlantic bluefin tuna tagging activity in the Mediterranean Sea has been based on conventional tags. These tags provide information on locations of release and recapture [30–33], and to date make up 10% of the more than 57,000 conventional tags on Atlantic bluefin tuna released between 1940 and 2003 [33]. More recently, several studies involving electronic bluefin tuna tagging have been carried out in the Mediterranean Sea. De Metrio *et al*. [20,21,34] tagged mature bluefin tuna in fishing traps and tuna farms with single-point pop-up satellite tags (PST) providing limited results due to some early technical problems with the power output of the Argos satellite transmitters in these first generation tags. A study from juvenile Atlantic bluefin tuna tagged with archival tags in a tuna farm in Croatia [35] focused on vertical movements of the fish in the central Mediterranean Sea. A more recent study, using early models of the pop up satellite archival tags (PAT), has confirmed the rapid migration of post-spawning adults from the western Mediterranean to the Atlantic [36]. Other studies have also recently highlighted the importance of the Mediterranean Sea habitats not only for spawning but also as an ecological foraging area significant during overwintering periods [37–42].

The spawning time for bluefin tuna occurs during June and July in the western and central Mediterranean Sea [18,30,43–47]. Frontal structures and anticyclonic gyres are associated to the spawning strategy of the mature fish in the Catalan Sea [48] and in the Balearic Archipelago [49,50]. In the Balearic Sea, high numbers of bluefin tuna larvae have been collected in tows often associated with waters with an oceanographic signature of Atlantic origin (i.e. Sea Surface Salinity (SSS) up to 37.2) or with areas where Atlantic and Mediterranean waters mix (i.e. SSS between 37.2 and 38); with warm surface waters, mostly ranging from 23.5 to 25°C [49,51,52]; and with geostrophic velocities ranging from 0.0168 to 0.37 m/s [49]. Bluefin tuna showed a preference for deep waters surrounding the Balearic Islands [49], and for Chlorophyll-a (Chl-a) concentrations ranging from 0.08 to 0.15 mg/m^3^ in their Mediterranean spawning habitat [53]. A well-defined thermocline, preferably having a negative gradient of 3°C, has also been hypothesized to be important for spawning [54,55]. Consistent with this location is the arrival and recapture of western tagged bluefin tuna in the Mediterranean Sea in these regions [13].

In the western Atlantic, studies with electronic tags reveal bluefin tuna have a significant preference in the Gulf of Mexico during the spawning season for continental slope waters (i.e. 2800 to 3400 m), moderate sea surface temperatures (SST) around 24–25°C or 26–27°C, surface current speeds from 0.126 to 0.316 m/s, and low surface Chl-a concentrations (0.10 to 0.16 mg/m^3^) [56]. Aggregation behavior is also evident in the tracks (a linearity index of 0.56 SD ± 0.13), and unique oscillatory diving behaviors has been described during the putative breeding phases in the Gulf of Mexico [12,25,56] and more recently, in the Mediterranean [36].

Following the recent studies that suggest extended residential behaviors of Atlantic bluefin tuna in the Mediterranean, the main objective of the present study was to better characterize the pattern for those individuals who remain residential in the Mediterranean even after spawning. To date there has been very little description of the diving behaviors or oceanographic preferences of the tuna in the Mediterranean Sea during the spawning period. Data on the migratory behaviors and other ecological features of the eastern Atlantic bluefin tuna tagged in the western and central Mediterranean were thus collected to be able to increase our knowledge on the how bluefin tuna utilize these habitats and the ecology within the Mediterranean all year round.

## Materials and Methods

### Electronic Tagging

**Fig 1:**
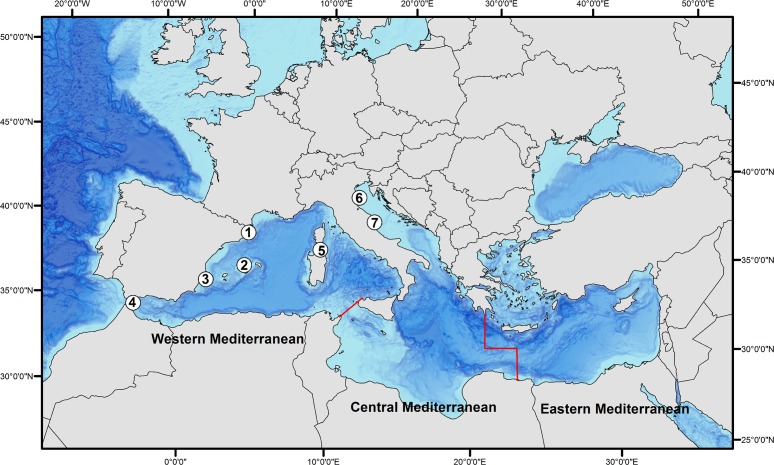
Numbers represent the different tagging locations (1. Roses/Llançà, 2. Pollença, 3. Moraira, 4. Algeciras, 5. Strait of Bonifacio, 6. San Benedetto del Tronto, and 7. Porto Barricata). Lines indicate the separation between the different Mediterranean basins based on FAO Fishing area criteria.

Atlantic bluefin tuna were tagged with electronic tags in expeditions that occurred between 2000 and 2012 in the western Mediterranean and the Adriatic Sea (Table 1). The first tagging expeditions were conducted in consecutive years of 2000 and 2001 in the Strait of Bonifacio (between Corsica and Sardinia), and the following expeditions in 5 consecutive tagging seasons from 2008 to 2012 in locations along the eastern Spanish coast—Roses and Llançà (NE Spain), Moraira (E Spain) and Algeciras (SE Spain)-, in the Balearic Islands—Port of Pollença (N Majorca, Spain)-, and in the Adriatic Sea—in San Benedetto del Tronto and Porto Barricata, E and NE Italy, respectively—(Fig. 1). Archival tags were only deployed in Roses (NE Spain). Tagging seasons started in the month of May (at the earliest) and ended by September. Tagging times depended on the accessibility of recreational fishers to bluefin tuna.

The research presented in this manuscript involved no endangered or protected species and no harm to the animals. The Stanford University Administrative Panel on Laboratory Animal Care research approved the protocol of the tagging operation in Corsica 2000 and 2001 (Protocol Number: 10786). No special permission was required for the other years in any of the locations as it was not required, however a special permit was granted by the Spanish Ministry "Agricultura, Alimentación y Medio Ambiente" for the 2011 tagging operations in Roses and Llançà (Table 1 and Fig. 1).

**Table 1:** Summary information on the deployments of electronic tags (>22 days on fish)—from 2000–2012 on western Mediterranean and Adriatic Sea bluefin tuna, analyzed in this study.

Year	Pop-up ID	Area	Deployment position	Deployment Date	CFL (cm)	Weight (kg)	Pop-off position	Pop-off	Days at liberty	Dart
2000	00–679	Corsica. France	41º19´N 09º17´E	9/9/2000	161	74.6	41º22´N 09º10´E	4/10/2000	25	Titanium
	99–581	Corsica. France	41º19´N 09º18´E	10/9/2000	134	43	41º27´N 08º57´E	3/11/2000	54	Titanium
	00–721	Corsica. France	41º18´N 09º18´E	10/9/2000	135	43.9	41º33´N 09º30´E	3/12/2000	84	Titanium
	00–735	Corsica. France	41º18´N 09º15´E	14/9/2000	131	40.1	42º40´N 04º40´E	6/12/2000	83	Titanium
	99–745	Corsica. France	41º19´N 09º18´E	14/9/2000	167	83.3	41º18´N 09º15´E	4/12/2000	81	Titanium
	00–720	Corsica. France	41º19´N 09º18´E	14/9/2000	142	51.1	41º08´N 10º04´E	6/11/2000	53	Titanium
2001	00–699	Corsica. France	41º19´N 09º30´E	12/9/2001	143	52.2	41º18´N 09º15´E	5/5/2002	235	Titanium
	00–731	Corsica. France	41º19´N 09º30´E	20/9/2001	152	62.8	41º18´N 09º15´E	5/1/2002	107	Titanium
	00–725	Corsica. France	41º18´N 09º25´E	20/9/2001	140	49	41º18´N 09º15´E	18/11/2001	59	Titanium
	99–716	Corsica. France	41º18´N 09º25´E	14/9/2000	137	45.9	41º17´N 09º23´E	6/12/2000	83	Titanium
2008	08A0398	Majorca, Spain	40º00´N 03º09´E	16/08/2008	-	100	37º88´N 01º74´E	15/10/2008	60	Umbrella
	08A0391	Majorca, Spain	40º00´N 03º09´E	16/08/2008	-	150	35º88´N 00º49´E	19/12/2008	125	Umbrella
	08A0393	Majorca, Spain	40º00´N 03 09´E	17/08/2008	-	150	38º96´N 00º 21´E	26/10/2008	70	Umbrella
	08A0405	Majorca, Spain	40º00´N 03º09´E	17/08/2008	-	50	40º26´N 04º29´E	29/10/2008	73	Umbrella
2009	08A0407	Majorca, Spain	40º01´N 03º01´E	14/08/2009	-	110	39º46´N 02º40´E	23/10/2009	70	Umbrella
	08A0390	Majorca, Spain	40º01´N 03º01´E	15/08/2009	-	65	39º58´N 03º37´E	14/09/2009	30	Umbrella
	08A0399	Roses, Spain	42º20´N 03º20'E	27/08/2009	160	73.2	38º09´N 14º00´E	7/12/2009	102	Umbrella
	08A0385	Adriatic	42º48´N 14º35´E	13/09/2009	-	45	43º46´N 13º42´E	25/12/2009	103	Umbrella
	08A0394	Adriatic	42º49´N 14º37´E	14/09/2009	132	41.1	30º38´N 19º02´E	5/3/2010	172	Titanium†
	08A0409	Roses, Spain	42º23´N 03º20´E	4/9/2009	-	45	38º58´N 05º19´E	17/12/2009	104	Prince
2010	08A0403	Majorca, Spain	40º02’N 03º10´E	8/8/2010	190	122.8	38º16’N 07º01’E	25/09/2010	48	Titanium†
	10P0049	Roses, Spain	42º15´N 03º40´E	1/9/2010	95	15.3	39º52’ N 6º20´E	11/11/2010	71	Titanium†
	10P0052	Roses, Spain	41º51´N 03º50´E	5/9/2010	96	15.7	37º48’ N 9º27´E	2/11/2010	58	Titanium†
	08A0390	Adriatic	42º55´N 14º14´E	13/09/2010	143	52.2	41º34’N 15º57’E	18/05/2011	247	Titanium†
	08A0396	Adriatic	44º49 N 12º51'E	24/09/2010	153	64	42º34’N 15º49’E	20/03/2011	177	Titanium†
2011	10P0406	Adriatic	43º01'N 14º09'E	26/07/2011	155	66.6	40°55'N 18°05'E	2/9/2011	38	Titanium†
	10P0400	Adriatic	43º01'N 14º09'E	26/07/2011	136	44.9	44°05'N 14°57'E	30/09/2011	66	Titanium†
	10P0038	Adriatic	42°57’N 14°16’E	6/8/2011	125	34.8	40°40'N 01°16'E	5/4/2012	243	Titanium†
	10P0401	Adriatic	42°56’N 14°17’E	6/8/2011	134	43	32°23’N 17°47'E	9/2/2012	187	Titanium†
	08A0389	Majorca, Spain	40º03'N 3º08'E	12/8/2011	177	99.2	39°58'N 02°57'E	30/09/2011	49	Titanium†
	10P0398*	Moraira, Spain	39º06’N 0º 29’E	29/05/2011	135	43.9	39°46'N 0°59'E	25/08/2011	88	Titanium†
	10P0402*	Roses, Spain	42º21'N 3º19'E	31/08/2011	144	53.4	37°48’N 10°36'E	30/06/2012	304	Titanium†
	10P0546*	Roses, Spain	42º20'N 3º09’E	1/9/2011	135	43.9	35°55'N 13°25'E	30/06/2012	303	Titanium†
	10P0547	Roses, Spain	42º20'N 3º20'E	1/9/2011	149	59.1	40°55'N 04°59'E	29/09/2011	28	Umbrellax1
	08A0388	Roses, Spain	42º20'N 3º20'E	1/9/2011	240	248.1	41°58'N 03°43'E	3/11/2011	63	Titanium†
	09P0412	Llançà, Spain	42º20'N 3º20'E	3/9/2011	199	141.2	37°09'N 04°29'E	26/09/2011	23	Titanium†
2012	10P0648* ^+^	Adriatic	43^o^03N 14^o^08'E	12/5/2012	135	43.9	43^o^17´N 16^o^4´E	7/11/2012	179	Titanium†

Notes: *Tags remained attached during the spawning season;

† two darts were used to anchor the tag;

^+^ recovered tags.

Bluefin tuna were caught using recreational rod and reel fishing. Most bluefin tuna, under 30 kg, were captured by trolling lures, while adults were caught chunking the water with sardines to attract them. From 2000 to 2001 and from 2010 to 2012, adult bluefin tuna were brought on-board using a lip hook placed in the most rostral position of the lower jaw and pulled from the water to a wet vinyl mat. In some cases, a plastic landing net was used. In 2008 and 2009 adult bluefin tuna were tagged in the water using an aluminum tagging pole with only one insertion point and tag anchor. Two types of darts were used during the tagging expeditions, a titanium dart and an umbrella plastic dart.

Bluefin tuna that were brought on board the vessel and had a soft cloth soaked in a fish slime replacement (PolyAqua, Novalek) placed over their eyes while a seawater hose oxygenated their gills. A clip of the pectoral fin was kept for future genetic analyses, and curved fork length (CFL) was measured to the nearest 0.5 cm. CFL was transformed to fork length [57] and then to weight using the formula adopted by ICCAT for Mediterranean tuna (Arena *unpublished* cited in [58]). For those tuna tagged in the water a conservative estimation of the weight was made by the tagging team.

Electronic tagging was conducted with Pop-up Satellite Archival Transmitting tags (PAT MK10 and in the last three years MiniPAT tags built by Wildlife Computers, Redmond Washington). All satellite tags were placed on juveniles and adults in the base of the second dorsal fin. In addition, archival tags (MK9, Wildlife Computers), were surgically deployed in juvenile bluefin tunas considered too small for an external satellite tag.

A total of 92 electronic tags were deployed during the duration of this study: 62 pop-up satellite tags and 30 archival tags. From the 62 pop-up satellite tags deployed, 37 (25 tags from 33 total deployments of MK10 and 12 tags from 29 MiniPAT deployments) transmitted data and were at liberty for more than 22 days, which was the minimum time that we used to be included in this work (Table 1). Several dart types were used including titanium, umbrella and “Prince” nylon darts, All darts were attached to the tag using a similar tether constructed of 130 kg monofilament and shrink-wrap. When bluefin tuna were brought on board the deck, a second attachment loop was used to prevent excessive motion of the pop-up satellite tag (except in the early 2000 and 2001 expeditions where only 1 dart was used, see Table 1). This second anchor point was built with a titanium dart using 81.6 kg monofilament covered with a shrink wrap cover. Pop-up satellite archival tags were programmed to release from the tuna between 150 and 300 days post deployment.

Pop-up satellite tags recorded pressure, light and water temperature every 60 seconds interval. In 2000 and 2001, for Corsica pop-up satellite tags, temperature and depth data were grouped in 24-hour binned histograms. Temperature layers were set at 6, 10, 12, 14, 16, 18, 20, 22, 24, 26, 28 and 60°C and depth layers (calculated from pressure) were -1, 5, 10, 50, 100, 150, 200, 250, 300, 500, 700 and over 1000 m. The 2008–2012 pop-up satellite tags were grouped in 6-, 12- or 24-hour binned histograms (for 2012, 2008 to 2010, and 2011 tags, respectively), and temperature layers were set at 3, 6, 9, 12, 15, 18, 21, 24, 27, 30, 33 and 45°C. In 2008 and 2009 depth layers data were set at 0, 10, 20, 50, 100, 150, 200, 300, 400, 500, 600, 700, 800, and over 800 m. From 2010 depth layers were set at 0, 2, 10, 20, 50, 100, 150, 250, 300, 400, 500, 2000 and over 2000 m. Pop-up satellite tag number # 10P0648 was returned by a fisherman, getting access to the 5-second interval archival record.

The 30 MK9 archival tags (i.e. 21 in 2008, 2 in 2009, 2 in 2010 and 5 in 2011) were surgically implanted in the peritoneal cavity of juvenile tuna and programmed to record pressure, light, external temperature and internal temperature every 60 s. To implant the archival tags, a 3–4 cm incision with a sterile surgical blade was carefully made in the ventral musculature of the tuna following the surgery methodology described in Boustany *et al*. [59]. Tags were labeled in English, Spanish and Japanese to provide information on the reward for returning the tag. A conventional green external tag (Floy Tags) was also inserted close to the second dorsal fin to inform about the existence of an archival tag inside the tuna.

Bluefin tuna geolocations were estimated from light level and sea surface temperature data recorded by the tags. All tracks were processed by CLS using a tool based in state-space models (SSM). SSM’s constitute a robust statistical approach to refine satellite tracking data by accounting for observation errors and stochasticity in animal movement. The algorithm uses sea surface temperature and bottom topography data to better constrain the tracks. An ensemble Kalman filter is applied to solve for the trajectory, thus estimating the state vector and its covariance from a set of samples rather than the usual deterministic equations [60–63]. The tags from the 2000–2001 expeditions were processed using a similar approach using custom algorithms as described in Teo *et al*. [64] and Block *et al*. [65].

FAO’s Major Fishing areas in the Mediterranean were adopted to separate the different basins [66] (Fig. 1).

### Potential Spawning Behaviors Recorded by Electronic Tags

Geolocation, depth and temperature data were analyzed for four tuna during the spawning season. From the 39 analyzed tuna, only these four had their tags attached during the breeding season and potentially were exhibiting behaviors associated with spawning tuna. The maximum, minimum and mean for the estimated SST, and the maximum bathymetry from each calculated position were calculated using the data provided by CLS.

To determine the movements of the four tuna, we calculated the linearity index (LI) [67]. LI is the result of the linear distance between the endpoints divided by the path distance for each fish. The LI was calculated grouping the data every 5–6 days.

Time at Depth (TAD, i.e. daily average of the percentage of time spent at different depth bins set up) and Time at Temperature (TAT, i.e. daily average of the percentage of time spent at different temperature bins set up) during the spawning season were only calculated for the pop-up satellite archival tag # 10P0398, while # 10P0402 and # 10P0546 did not transmit this information.

The only pop-up satellite tag recovered from the four tuna showing potential spawning behavior was from a presumed mature tuna tagged in the Adriatic (# 10P0648). The record provided data in a time series every 5 seconds and was analyzed looking for unique behaviors potentially in association with spawning. Depth records < 0 m were assumed to be zero and 12 bins limits, focusing on the first depth layers, were defined by 0, 2, 5, 10, 15, 20, 30, 40, 50, 70, 100, 200 and > 200 m using Matlab (Mathwork, v 7.13.0.564). Following the methodology described in Jorgensen *et al*. [68], we calculated a distance matrix based on a hierarchical cluster tree using an un-weighted average distance (UPGMA) linkage algorithm (‘linkage’ function in Matlab) to differentiate dive behavior modes through the time at liberty based on the vertical distribution (‘pdist’ function). Distance calculations were obtained using the ‘City block’ measure, where bin values <0.1 were assigned as zero to avoid outliers or rare events. Then, the clusters were plotted along with a dendogram using the Matlab ‘dendogram’ function. Also IGOR Pro 6 (WaveMetrics) was used to show detailed behavior per day. Dive frequency was calculated identifying individual dives per day and grouped by season and cluster. A dive begins when the fish starts descending, and each dive includes a descent and an ascent. A dive ends when the fish starts to descent a second time, so every valley between two peaks is considered a dive.

### Bluefin tuna fishing operations

To better understand habitat utilization of the bluefin tuna released in the Mediterranean Sea and for comparison with the electronic tagging data, the precise locations of the bluefin tuna fishing operations that had occurred inside the Mediterranean Sea from 1989 to 2012 were obtained (Fishspectrum company), for the period April to August. The dataset included purse seiners, longliners and bait boats from Algeria, Croatia, Egypt, France, Greece, Italy, Korea, Libya, Malta, Morocco, Spain, Tunisia and Turkey. In total, 6031 locations were established. Additionally, we added to these locations the spatial distribution of the Japanese longliners’ total bluefin tuna catches in Algerian waters from 2000 to 2007 [69].

### Oceanographic Analyses

Bluefin tuna tracks were analyzed in combination with available satellite oceanographic data, which provided information on the main oceanographic features in the Mediterranean Sea areas. The movement paths of the Atlantic bluefin tuna were overlaid on ocean bathymetry to visualize the association of the movements with the continental shelf and slope using a Global Self-consistent, Hierarchical, High-resolution Shoreline (GSHHS) database version 2.2. (http://www.ngdc.noaa.gov/mgg/shorelines/data/gshhs) and ETOPO2 2-minute global bathymetry/topography high-resolution database (http://rda.ucar.edu/datasets/ds759.3/).

The moderate resolution imaging spectroradiometer MODIS AQUA L3 satellite data (incorporating standard atmospheric corrections) were acquired with a resolution of 1.2 km at high temporal frequency (http://oceancolor.gsfc.nasa.gov/DOCS/MODISA_processing.html). We used the global 8-day mean Nighttime Sea Surface Temperature (NSST) grids from MODIS that had been validated (ftp://podaac.jpl.nasa.gov, thermal IR SST, 4 km and 0.1°C resolution). Additionally, we obtained averaged weekly daytime measures of Chl-a concentration from MODIS databases. For each tag location, we extracted a Chl-a value. SSS and Geostrophic Velocity (UV) (zonal and meridional currents) data were downloaded from MyOcean [70]. The NSST, Chl-a, SSS and UV raw data were used as auxiliary environmental data.

### Home range and habitat use

A utilization distribution (UD, [71]) was subsequently created from the satellite tracking using the ‘adehabitat’ package for R [72]. We used the default method for the estimation of the smoothing parameter, which supposes that the UD is bivariate normal. Under the utilization distribution model, we considered that the animals’ use of space can be described by a bivariate probability density function, the UD, which gives the probability density to relocate the animal at any place according to the coordinates (x, y) of this place. The home range deduced from the UD as the minimum area on which the probability to relocate the animal is equal to a specified value. For example, the 95% home range corresponds to the smallest area on which the probability to relocate the animal is equal to 0.95. Positions grouped by diving pattern (clusters) were represented using a Kernel density spatial analysis.

Statistical analyses for the oceanographic characteristics and the utilization areas were performed with the Statistics Toolbox in Matlab 8.0.0 (MATLAB 2011).

## Results

The mean size of the Atlantic bluefin tuna released in the western Mediterranean Sea with pop-up satellite tags was 76.1 kg± 50.1 (SD). The bluefin tuna tagged in waters off eastern Spain and the Balearic Islands were on average 88 kg ± 58.7 (SD) and Corsica fish were 54.6 kg ± 14.4 (SD). In the Adriatic Sea, the size of tagged tuna was lower (49.1 kg ± 10.6 SD). Overall, the mean weight of the satellite tagged bluefin tuna was 69.5 kg ± 45.2 SD, and only 7 of the 37 tagged tuna analyzed during the study were over 100 kg (the average of these 7 large tuna was 146 kg, ± 49 SD).

**Fig 2:**
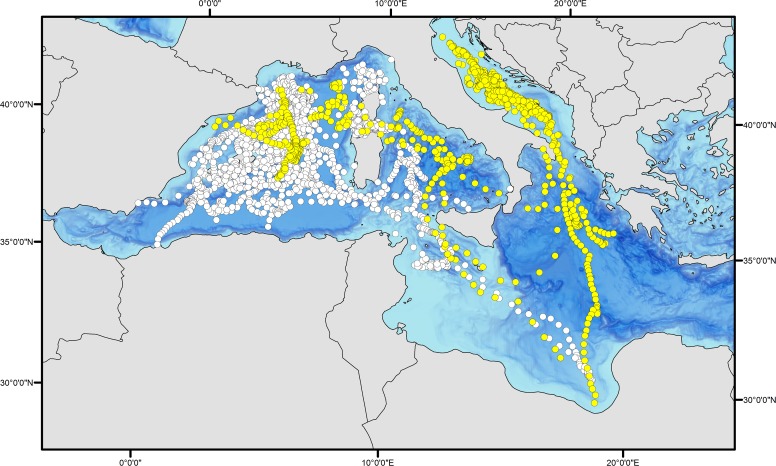
Daily positions based on estimated geolocation of the 38 tuna analyzed in this study and tagged within the Mediterranean Sea with pop-up satellite and archival tags. White circles represent the positions of the tuna tagged in the western Mediterranean basin (n = 28 pop-up tags + 1 internal tag), while yellow circles represent the positions of the tuna tagged in the Adriatic (n = 9 pop-up tags).

The mean retention time of the pop-up satellite archival tags from fish that reported more than 22 days (Table 1) was 81.5 ± 45.4 (SD) days when using single point attachment methods (N = 20). Bluefin tuna tagged on deck with the addition of a second attachment loop (N = 17) averaged 136.2 ± 96.1 (SD) days. Even though the retention time increased by using a second loop, only two of the pop-up satellite tagged fish with a second loop, remained attached until their programmed release date recording 303 and 304 days of data respectively (Table 1). All of the satellite tagged bluefin tuna remained within the Mediterranean Sea post release and throughout the track duration (Fig. 2). These western and central Mediterranean tagged bluefin tuna primarily resided within the basin they were released in, and none moved into the eastern Mediterranean basin.

**Fig 3:**
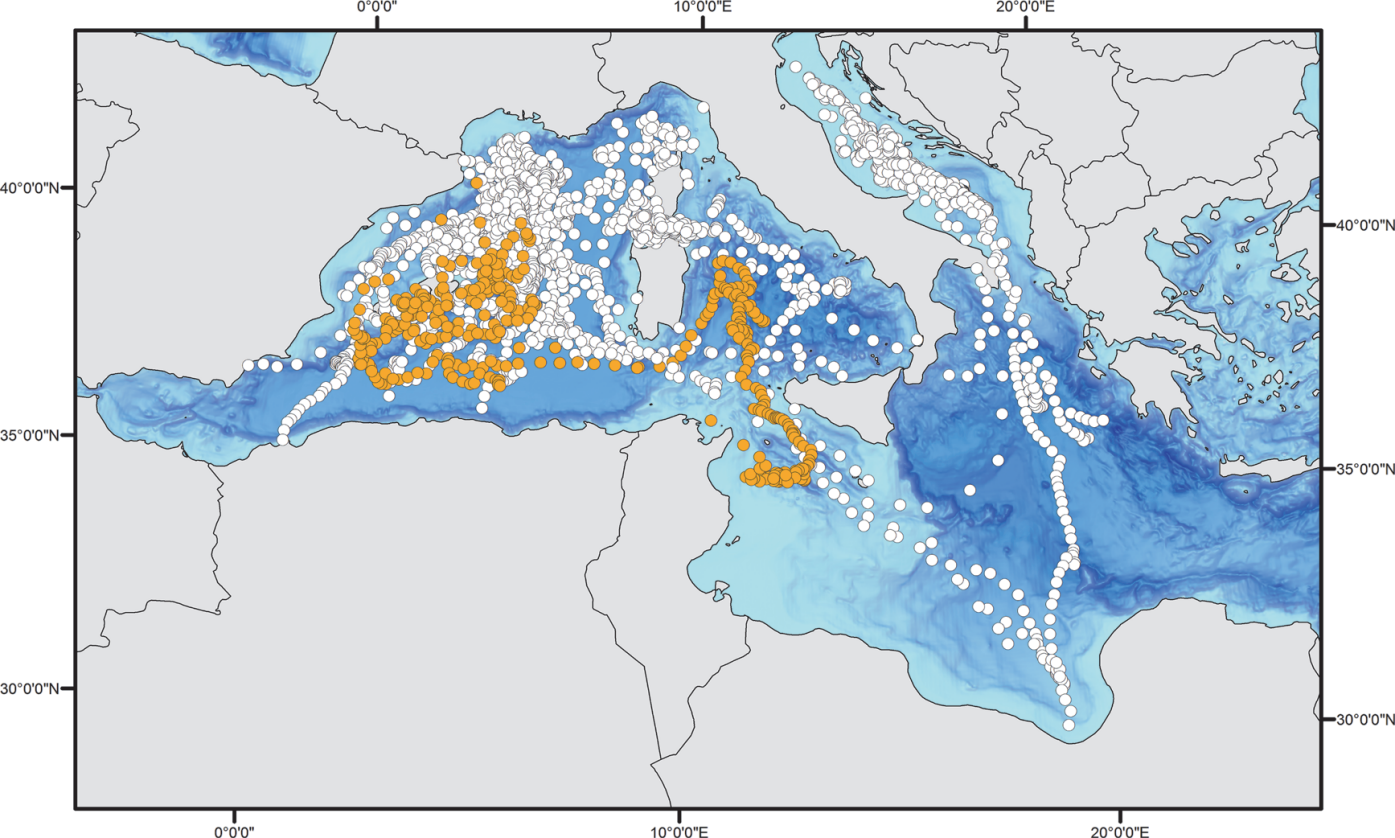
Daily positions for the 38 tuna analyzed in this study. Orange circles represent the internally tagged fish, while the white circles are the positions of the tunas tagged with pop-up satellite tags in both basins.

**Fig 4:**
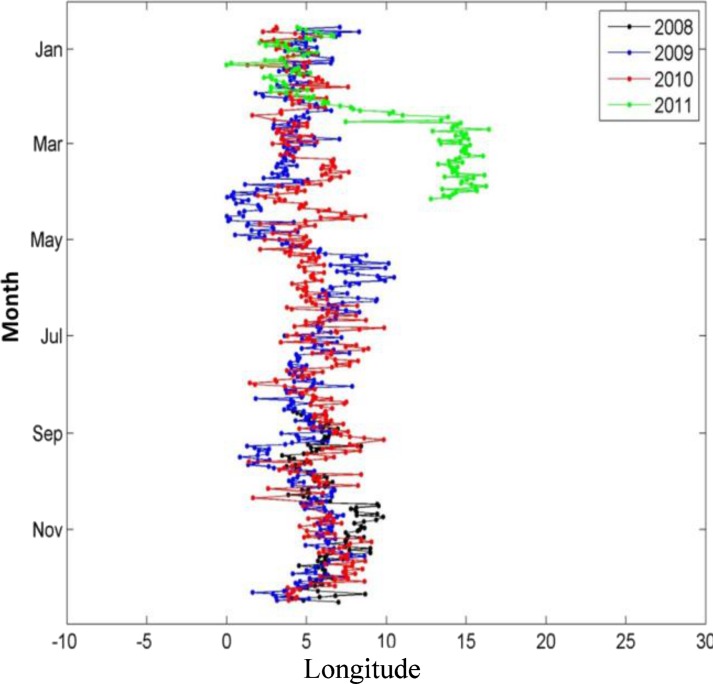
Longitude for internal tag #0890152 by year.

Two bluefin tuna estimated at 13 kg (body size at tagging) have been recovered from the 30 surgically implanted internally tagged bluefin tuna in the Mediterranean Sea. Both of these fish were tagged and released in August 2008 in the waters off Roses, NE Spain (Table 2). The first internal tag recovered was from a bluefin tuna recaptured by a Spanish pelagic longliner after 391 days at liberty (ID # 0890138). The fish was 185 km from its deployment location when recaptured over a year (Fig. 3). The second internal tag (ID # 0890152) was recovered by a fishmonger in Venice, Italy from a tuna that had been caught at an unknown position around western Sicily (after 965 days at liberty). The first tag provided a complete archival time series record; however upon recalibration of the latter archival tag, the external temperature sensor showed a deviation from the original calibration and its thermal data could not be taken into account in the analyses. Although the complete archival tag geolocation track is not available, we were able to use the light level longitude record to establish the position and concluded this bluefin tuna did not leave the Mediterranean, nor did it visit the eastern Mediterranean. This bluefin inhabited waters between the 0 and the 18^th^ meridian east (Fig. 4).

**Table 2:** Deployment data and recapture information of the implanted internal tags deployed in 2008 in Roses, NE Spain.

Year	Tag ID	Area	Deployment position	Deployment date	Weight (kg)	Recapture	Recapture position	Days at liberty
2008	890138	Roses. Spain	41º 56´N 03º 36´E	31/08/2008	12,1	26/09/2009	41° 01´N 02° 45´E	391
2008	890152	Roses. Spain	41º 56´N 03º 36´E	31/08/2008	13	23/4/2011	37° 30´N 11° 30´E	965

In total, the 38 reporting electronic tags (including internal tag # 0890138) recorded 4336 days of location and behavior data on bluefin tuna behavior and habitat utilization within the Mediterranean Sea. Electronic tags deployed on Atlantic bluefin tuna in waters off eastern Spain (N = 18) recorded a total of 1669 days (average 92.7 ± 81.2 per track) and 391 days for the internally tagged juvenile released in eastern Spain. The Corsica deployments (N = 10, all MK10 pop-up satellite tags) generated 864 days of data (average 86.4 ± 57 days per track), while the tags deployed in the Adriatic Sea (N = 9) recorded 1412 days (average 156.9 ± 73.2 per track). Combining both the satellite and internal tagging data sets (including the internal tag # 0890152) revealed that none of the fish analyzed during the present study left the Mediterranean Sea during the 23 to 964 days that they carried an electronic tag. The tagged bluefin tuna released in western or central Mediterranean locations stayed within these two basins, in some cases crossing between them, and never ventured into the Alboran Sea (the southwestern most area of the Mediterranean Sea, just before the Strait of Gibraltar) or the eastern Mediterranean basin.

### Habitat Use of Electronic Tagged Bluefin in the Mediterranean Sea

**Fig 5:**
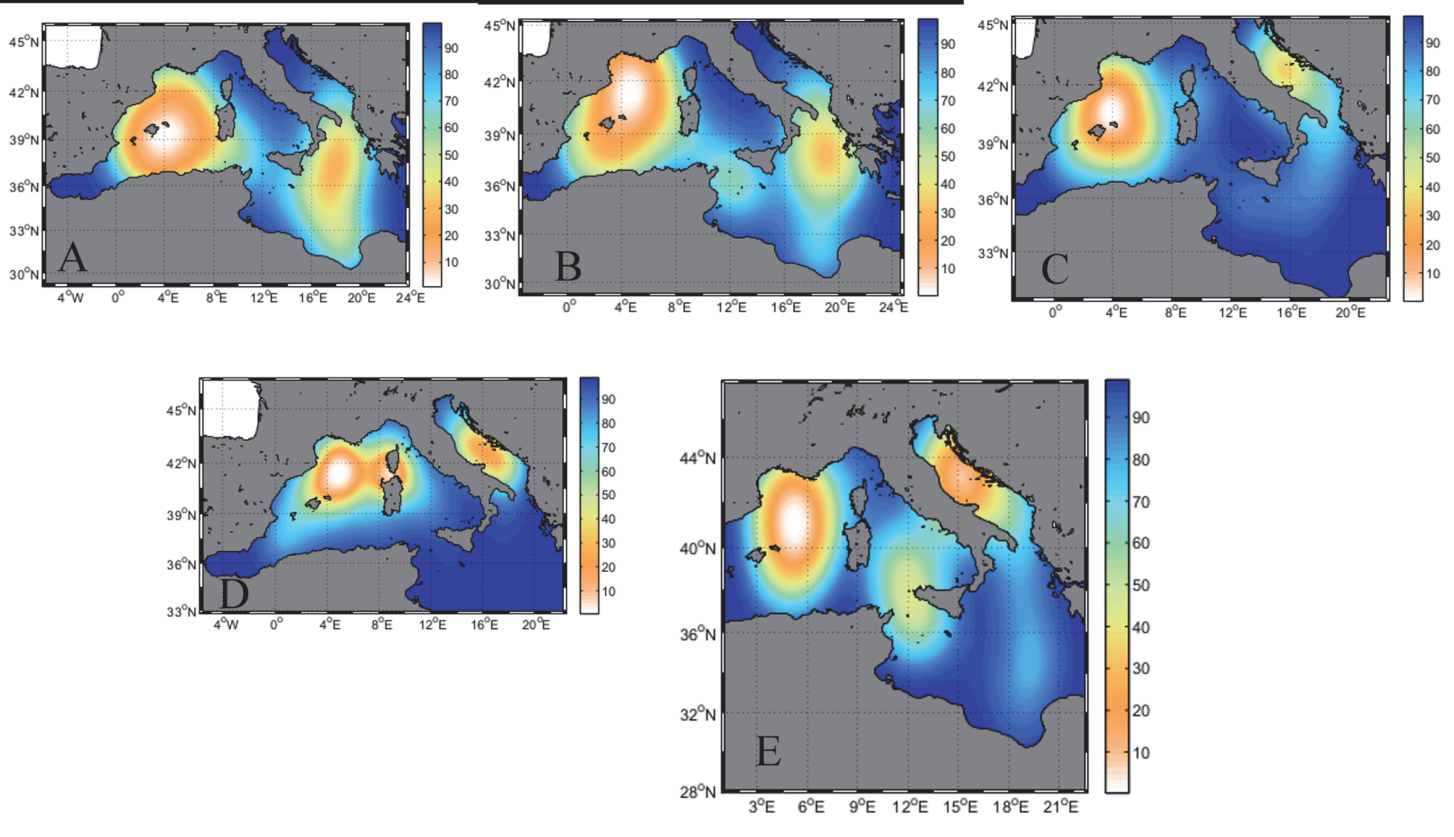
Seasonal Utilization Distributions (UDs) of bluefin tuna. The locations of the tuna were examined for the (A) spawning period (i.e. May 15 to July 15) and the seasonal periods of (B) spring, (C) summer, (D) autumn and (E) winter. UDs were computed using the Kernel method through the ad hoc method (h = Sigma*n^(-1/6), Sigma = 0.5*(sd(x)+sd(y))). The graphs show the UDs cumulative frequencies up to 95% (the color attributed to a given percentage p applies to areas comprised between p and p–1% isopleths).

The seasonal analysis of the oceanic habitat use of the 38 tunas included in the analyses (Fig. 5) clearly showed the waters around the Balearic Islands and between the Ionian Sea and Libya are of high importance. The waters around the southern part of the Balearic archipelago were key waters occupied during the putative spawning period (Fig. 5A). Moreover, the main overwintering/feeding areas were identified as the waters south of the Gulf of Lions (Fig. 5B-5E) and the Adriatic Sea, primarily in this latter region the deeper waters of the Jabuka Pit (Fig. 5C-5E).

The trajectories of all the tagged tuna analyzed here, showed a distinct use of the basins within the Mediterranean (S1–S3 Figs).

### Adriatic

In total, 800 days of location and behavioral data (diving) were recorded inside the Adriatic Sea from the 9 pop-up satellite tags deployed there, 288 days in the western Mediterranean Sea basin (including the Tyrrhenian Sea), and 150 days in the Ionian Sea as well as in waters close to Libya. Geolocation position data demonstrated that tuna under 60 kg left the Adriatic Sea, while those over 60 kg (N = 2, Table 1) remained in the Adriatic Sea. The trajectories of these Adriatic Sea electronically tagged bluefin tuna show a distinct use of the regional basins within the Mediterranean Sea (S1 Fig.). Tuna tagged and released in the Adriatic Sea displayed an association with the waters over the bathymetrically deeper areas of this region, particularly the Jabuka Pit/Fossa di Pomo (i.e. mid-Adriatic) and the South Adriatic Pit, during all seasons. These fish showed a strong residency in these deeper bathymetry waters areas of the basin during all seasons except for spring—when most tags had already detached—(Figs. 2 and 5). Only two of these nine bluefin tuna left this central Mediterranean region towards the end of September to venture into the western portion of the Mediterranean Sea (ID # 10P0038 and 10P0401, S1.7 and S1.8 Figs.). These two fish, moved into the northwestern Mediterranean regions (in the vicinity of the Gulf of Lions), visiting the waters surrounding Corsica and Sardinia Islands on the way. After spending some time around Corsica and Sardinia and in the Tyrrhenian Sea, one of these individuals moved towards Libyan waters at the end of January where its satellite tag popped-off and reported on February 9—before the known spawning season. Two additional tuna tagged in the Adriatic Sea (ID #08A0394 and 08A0390, S1.2 and S1.3 Figs.) left the deployment area—although they remained within the central Mediterranean basin—and visited the Ionian Sea (between mid-March and beginning of May) and the Gulf of Sidra (from mid-January to the beginning of March) (Figs. 3, 5B and 5E).

From the nine Adriatic tuna, only one was tagged before the spawning season (i.e. # 10P0648, Table 1 and S1.9 Fig.). This fish left the Adriatic Sea towards the end of May, spent time in the Ionian Sea and in the waters south of Sicily, and came back to the Adriatic Sea around mid-August.

### Western Mediterranean

Western Mediterranean electronic tagged adult tuna showed more fidelity to their deployment basin than the Adriatic ones (S2 Fig.). Even though the average of time at liberty of their deployments (i.e. 90.6 ± 72.4 (SD) days) limited the dispersion of the fish and could have biased the general view of their movements, overall, these tuna tended to concentrate in presumed feeding grounds (i.e. in the waters between the Gulf of Lions and the Balearic Islands) all year round (Figs. 5B to 5E), and only one individual went through the Strait of Sicily (i.e. # 10P0546, Table 1), reaching the Gulf of Sidra in June, during the known spawning season (Fig. 5A, S2.14 Fig.). A second tuna (i.e. # 10P0402, Table 1, S2.15 Fig.), was at the entrance of the Strait of Sicily when its tag released on its programmed day.

The two tuna with the longest duration of pop up satellite tag deployments (303 and 304 days, i.e. # 10P0546 and 10P0402, respectively), were deployed in Roses, NE Spain, and both occupied the aforementioned feeding area between the Gulf of Lions and the waters north of the Balearic Islands. This area is similar to that occupied by a bluefin tuna tagged in the Adriatic Sea (i.e. # 10P0038, Table 1 and S1.7 Fig.). This regional hot spot occurs from November until March (Figs. 2, 5 and S2.14 and S2.15 Figs.).

The juvenile Atlantic bluefin tuna tagged with an internal tag (# 0809138, Table 1) also displayed a different behavior from the larger sub-adult and adult tuna occupying the southern waters of the western Mediterranean basin. From the end of autumn to the beginning of spring this tuna visited the Tyrrhenian Sea and crossed the Strait of Sicily. From there it returned to the Balearic spawning ground and spent the rest of spring and summer in the waters around the Balearic Islands, until it was recaptured at the end of September, after 391 days at liberty (Fig. 3, S2.19 Fig).

Corsican individuals (N = 10), in particular, showed a high fidelity to the Strait of Bonifacio and around the northern area of Corsica (Fig. 5d, S3 Fig.).

### Bluefin Tuna Fishing Operations

**Fig 6:**
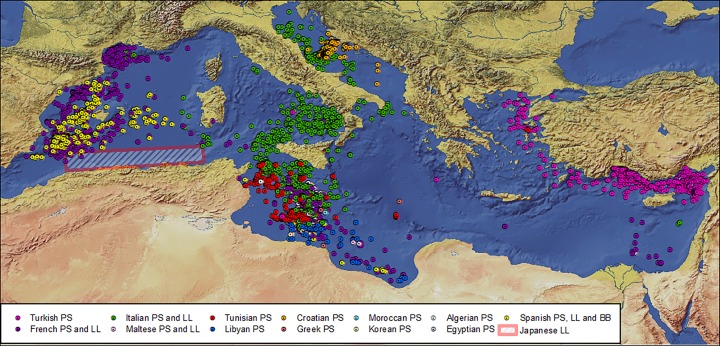
Locations of bluefin tuna fishing operations between April and August in the Mediterranean Sea from 1989 to 2012 (n = 6031) (different color dots), and spatial distribution of the Japanese longliners’ total bluefin tuna catches in Algeria from 2000 to 2007 (based on Abdelhadi *et al*.,[69]) (red dotted line). PS = Purse seiners, LL = Longliners and BB = Bait boats.

All the fishing operations (N = 6031) that occurred within the Mediterranean Sea, from 1989 to 2012, from purse seiners, longliners and bait boats were plotted together to examine potential aggregation sites of fish (Fig. 6).Altogether these catch data locations provide a proxy for the bluefin tuna distribution inside the Mediterranean during the past 23 years from April to August, as fishing vessels (mostly purse seiners coupled with spotting planes that were helping the fishery until recently) target mainly those areas where bluefin tuna aggregate.

Comparing the fishing operations’ map with the trajectories and habitat use distributions of the 38 tags analyzed in the present study (Figs. 3, 5 and S1–S3 Figs.), it can be observed that the fisheries data and the tagging data overlap almost completely; putting aside the Gulf of Naples (Tyrrhenian Sea) and the Levantine Sea, which were never visited by the electronic tagged tunas. Taken together the two data sets provide the likely geographical distribution of the species preferred habitats in the Mediterranean Sea and their potential foraging and reproductive hot spots.

### Habitat Use and Behavior During the Spawning Season

**Fig 7:**
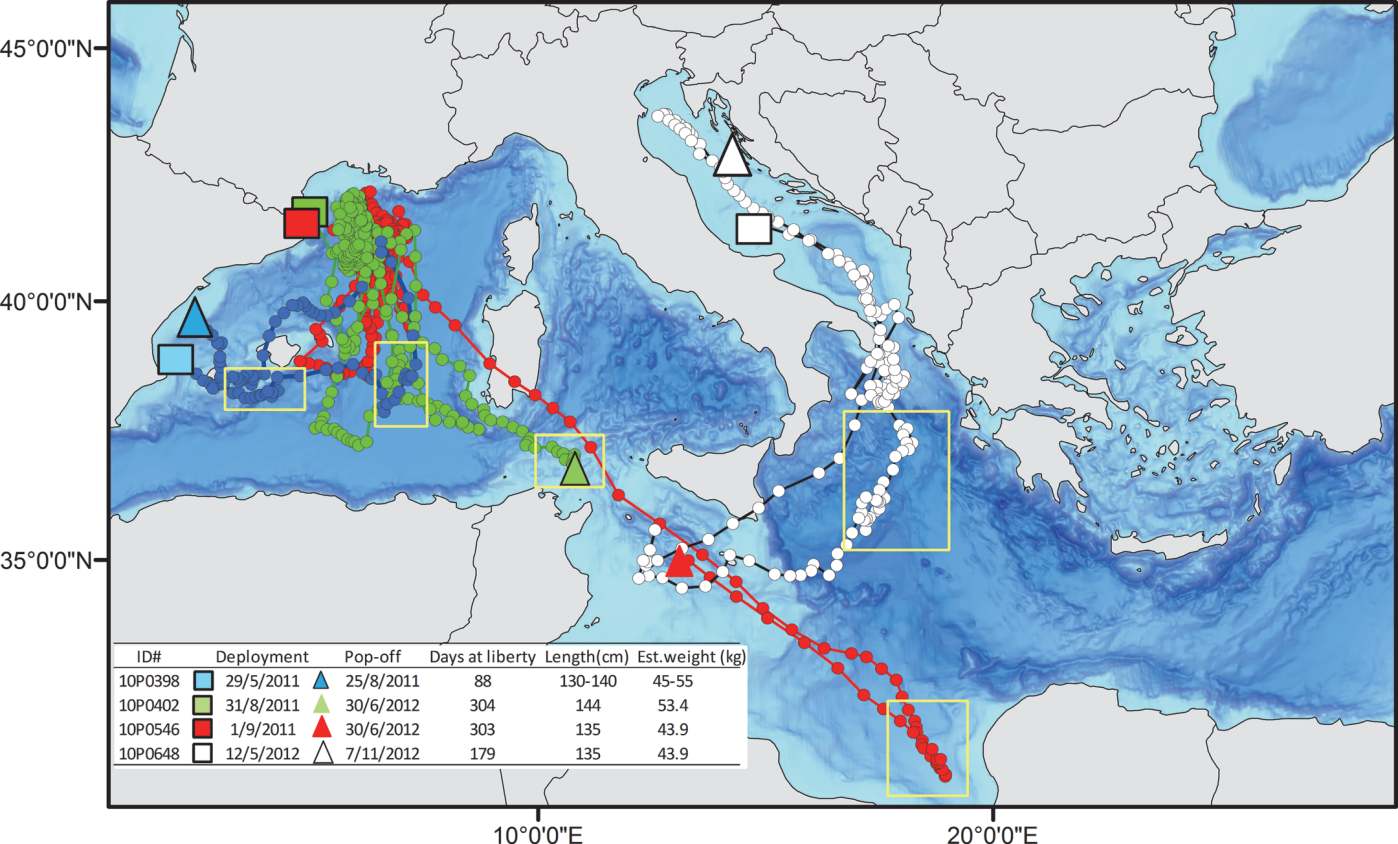
Tagged bluefin tuna possibly engaging in spawning during the reproductive season. Note: yellow squares indicate periods of time with low linearity values and possible spawning behavior.

Atlantic bluefin tuna are known to spawn in the Gulf of Mexico and Mediterranean, which provides the first indications of the locations, oceanographic parameters and behavioral patterns observed during the spawning period (see Introduction). Based on prior electronic tagging in the western Atlantic and the Mediterranean, specific patterns, including aggregation behavior in positional data, a period of shallower mean depths from diving records, coupled to a unique oscillatory diving pattern, and warm ambient temperature ranges have been observed during the spawning seasons of both, the Gulf of Mexico and the Mediterranean [25,36,49,56]. Only four of the bluefin tuna in this study, were at liberty with their tags attached during the known Mediterranean spawning season and considered at this time by size to be potentially mature adults (tags # 10P0648, 10P0398, 10P0402 and 10P0546) (Fig. 7). Variance statistics of the daily estimated positions for the trajectories of these four tunas can be found in S1 Table.

**Fig 8:**
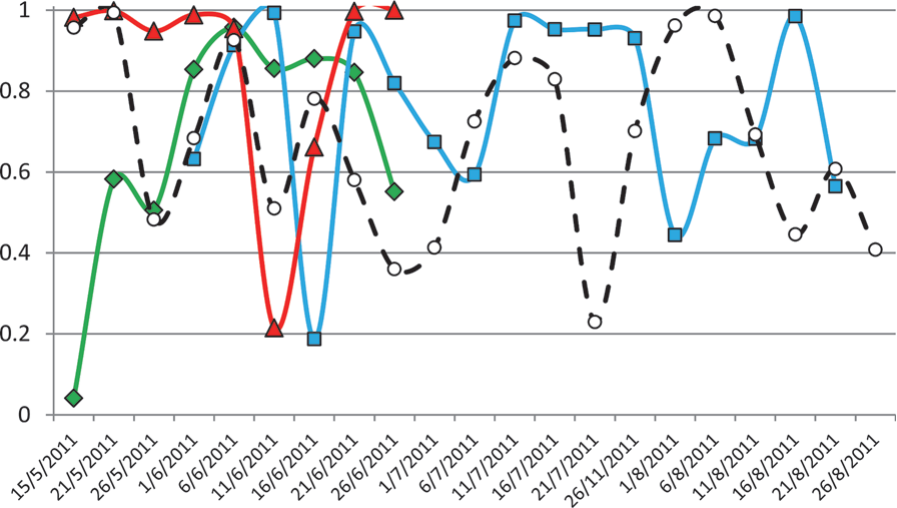
Linearity index for the four tags at liberty from mid-May to October. Green #10P0402, red #10P0398, black #10P0546 and blue #10P0648.

This bluefin tuna with tag # 10P0648 (43.9 kg and 135 cm CFL) that potentially could be classified as a Mediterranean spawner, was tagged in the Adriatic Sea on May 12, 2012 (Fig 7). The fish left the Adriatic on the 25^th^ of May, returning to it on September 9 after 107 days at large outside the Adriatic. The pop-up satellite tag was recovered after 179 days close to the coast of Croatia in November 2012. A full archival data record (pressure, temperature and light time series) was obtained with an interval of 5 seconds providing a high resolution time series. The linearity index (LI) score showed residency periods along the track in different areas of the Ionian Sea (Fig. 8). The first period of residency occurred from the 26^th^ to the 31^st^ of May just after leaving the Adriatic (LI 0.48 and SST 19.79°C SD ± 0.38), a second period of aggregation occurred from the 11^th^ to the 15^th^ of June (between Zakynthos Island, Greece, and the southernmost point of continental Italy), with a Linearity index of 0.51 and SST of 23.63°C (SD ± 0.26), followed by 15 consecutive days of low LI (0.58, 0.36 and 0.41 for the periods 21^st^ to 25^th^, 26^th^ to 30^th^ of June and 1^st^ to 5^th^ of July, respectively; while SST for the same periods was 25.64°C (SD ± 0.50), 26.19°C (SD ± 0.30) and 27.31°C (SD ± 0.19), respectively). The next resident behavior period occurred in Tunisian waters late in July (LI of 0.23 and SST of 25.44°C (SD ± 0.61) for the 21^st^ to the 25^th^ of July). Average bathymetry for the waters occupied for the period of the 26^th^ to the 31^st^ of May was 1091.29 m (SD ± 126.50), while for the June periods and the first period of July the average was 3511.41 m (SD ± 120.23). The bathymetry shoaled during the residential event of the 21^st^ to the 25^th^ of July (413.28 m, SD ± 425.90).

**Fig 9:**
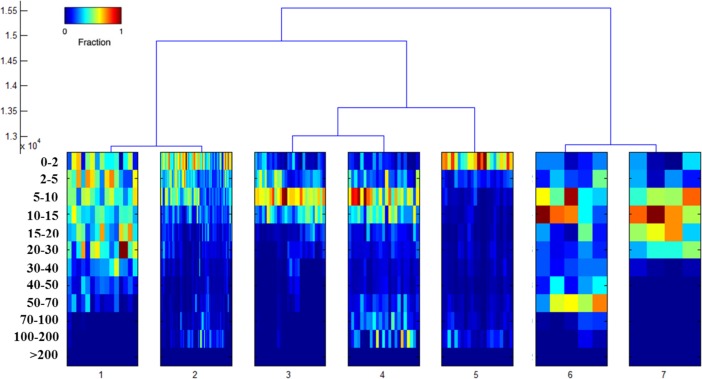
Dendrogram for tag #10P0648 determined from clustering analysis of differences in diving patterns Each column represents a 24-hours depth histogram (n = 175 days) and the color is the fraction of time. The density variable is expressed as a fraction of each day spent in depth bins defined along the y-axis.

**Fig 10:**
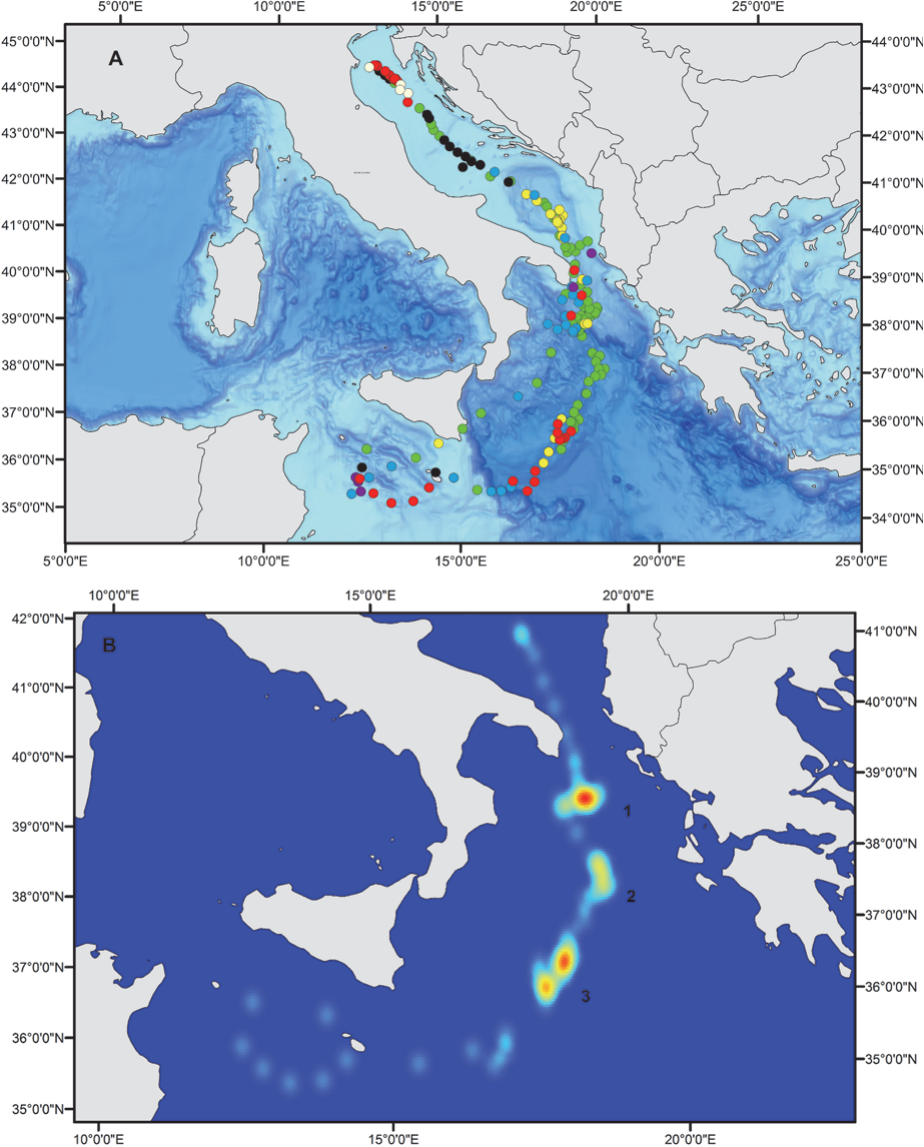
(A). Positions of the different clusters along the track of the tuna 10P0648 (in Black cluster #1, green cluster #2, red cluster #3, blue cluster #4, yellow cluster #5, violet #6 and in white cluster #7). (B) Kernel distribution of cluster 2 and 3 during May, June and July. Number #1 compiled the period from 25^th^ May to 5 ^th^ June, #2 from 7th to 14th June while #3 is for 17th to 30th of June.

To examine the diving behavior and depth distribution of this fish (# 10P0648) in more detail we performed a cluster analysis. This revealed 7 types of diving behavior modes (Figs. 9, 10A and Table 3). All 7 groups are described herein. The depth-bin histogram plot for cluster 2 (43.60% of the track) showed a higher preference of time in the surface waters, higher in use from the surface to 2 m and 5 to 10 m bins during the period from May to June (Fig. 10). Mean depth for cluster 2 for all days at liberty was 38.02 m (SD ± 41.47), while the average external temperature was 20.50°C (SD ± 1.86) and the dive frequency 2011 (SD ± 343). Maximum “U”-shaped dives down up to 300 m were observed in this cluster. The cluster 2 was predominant and mostly concentrated during June (14.53%) followed by May (8.72%), August (8.13%), September (6.39%) and October (4.06%). The mean depth varied during the months during the described spawning season. Values ranged from 14.58 m (SD ± 4.76) in May, 19.25 m (SD ± 17.10) in June and 30.51 m (SD ± 21.00) in July, while it continued increasing from August to September (Table 4), where there was no bathymetry limitation, and decreased again from September to October when moving through the North Adriatic basin where a shallower bathymetry was evident. External temperature means recorded by the tag were 17.85°C (SD ± 0.63), in May, 21.54°C (SD ± 1.65), 21.97°C (SD ± 2.38) and 21.28°C (SD ± 1.05) for June, July and August (Table 3), maximum external temperature (24.68°C) was reached in July.

**Table 3:** Mean values, maximums, minimums and standard deviation for the clusters obtained.

	Mean				Mean				Mean			
	Depth	Min	Max	SD	E. Temperature	Min	Max	SD	Dive Frequency	Min	Max	SD
Cluster1	16.77	11.95	21.46	2.71	20.42	16.85	23.58	1.49	1694	841	2365	321
Cluster2	38.02	5.00	148.58	41.47	20.50	16.83	24.68	1.86	2011	1374	3076	344
Cluster3	10.35	5.70	17.62	3.15	22.19	17.94	26.09	2.49	1765	1486	2171	190
Cluster4	39.20	17.85	109.42	25.09	21.30	17.09	23.28	1.45	1550	1248	1978	205
Cluster5	49.16	8.54	112.02	33.11	21.46	19.26	24.09	1.50	2586	1835	3352	431
Cluster6	26.11	18.93	32.70	5.09	20.69	19.08	22.77	1.49	1788	1553	1972	151
Cluster7	12.91	11.98	13.96	1.00	20.61	19.51	21.27	0.79	1674	1455	1857	177

**Table 4:** Mean values, maximums, minimums and standard deviation for cluster 2 and 3 for all months when present.

		Mean				Mean				Mean			
	Month	Depth	Min	Max	SD	E. Temperature	Min	Max	SD	Dive Frequency	Min	Max	SD
Cluster 2	May (n = 15)	14.58	8.00	23.07	4.76	17.85	16.83	18.87	0.63	1977	1628	2126	157
	June (n = 25)	19.25	5.00	86.03	17.10	21.54	19.92	24.54	1.65	2195	1646	3076	346
	July (n = 3)	30.51	15.45	54.46	21.00	21.97	20.17	24.68	2.38	2287	2158	2415	128
	August (n = 14)	59.93	22.48	129.66	42.26	21.28	20.16	23.55	1.05	1766	1439	2197	273
	September (n = 11)	104.32	19.80	148.58	42.54	20.26	19.26	22.28	1.00	1751	1374	2079	243
	October (n = 7)	10.37	2.62	13.41	2.43	20.71	19.53	21.25	0.62	2313	2011	2654	253
Cluster 3	June (n = 5)	7.70	5.70	10.46	2.01	23.45	20.40	25.06	1.91	1863	1660	2126	185
	July (n = 11)	12.47	7.12	17.02	3.79	24.17	21.32	26.10	1.63	1760	1486	2171	205
	October (n = 7)	9.70	7.46	11.29	1.22	20.20	19.28	21.09	0.44	1719	1492	2019	192

Cluster 3 pattern from this analyses corresponded to a high utilization of the water column comprised between 5 and 10 m with very few deep dives. Utilization of the surface waters (bins 0–2 m) was scarce. This behavior corresponded to the 16.20% of the entire track and was found in May (1.16%), June (2.9%), July (6.30%) and October (5.81%). Average depth of cluster 3 was 10.35 m (SD ± 3.15), external temperature 22.19°C (SD ± 2.49) and dive frequency 1765 (SD ± 190) (Table 3). June average depth was 7.70 m (SD ± 2.01), July 12.47 m (SD ± 3.79) and 9.70 m (SD ± 1.22) in October where the dive depths were bathymetrically constrained (Table 4). Cluster 4 (13.37%) showed a similar diving pattern on the first meters (0 to 15 m) as cluster 3 but showing increased residency between 100 and 200 m (Table 3). Cluster 5 (12.79%) corresponded to a surface behavior (bin 0 to 2 m) with occasional residency between 100–200 m. This behavior was found especially in the month of September around the South Adriatic Pit. Cluster 6 behavior (2.9%) was showed only during 5 days when visiting shallow waters (Table 3).

The last groups, cluster 1 (8.7%) and 7 (2.3%) mainly corresponded to the Adriatic basin (October) or other areas with a limited bathymetry, like the Tunisian basin (July). Here the shelf limits the diving of the tuna. The tuna exploited the entire water column in this region, but cluster 1 primarily used the upper layers (0–5 m). Maximum depth bins observed were 100 to 200 m for cluster 1 and 30 to 40 m for cluster 7. The mean depth was 16.77 m (SD ± 2.71) and 12.91 m (SD ± 1.00) for clusters 1 and 7, while external temperature and dive frequency were 20.42°C (SD ± 1.49), 20.61°C (SD ± 0.79) and 1694 dives (SD ± 321) and 1674 dives (SD ± 177), respectively.

**Fig 11:**
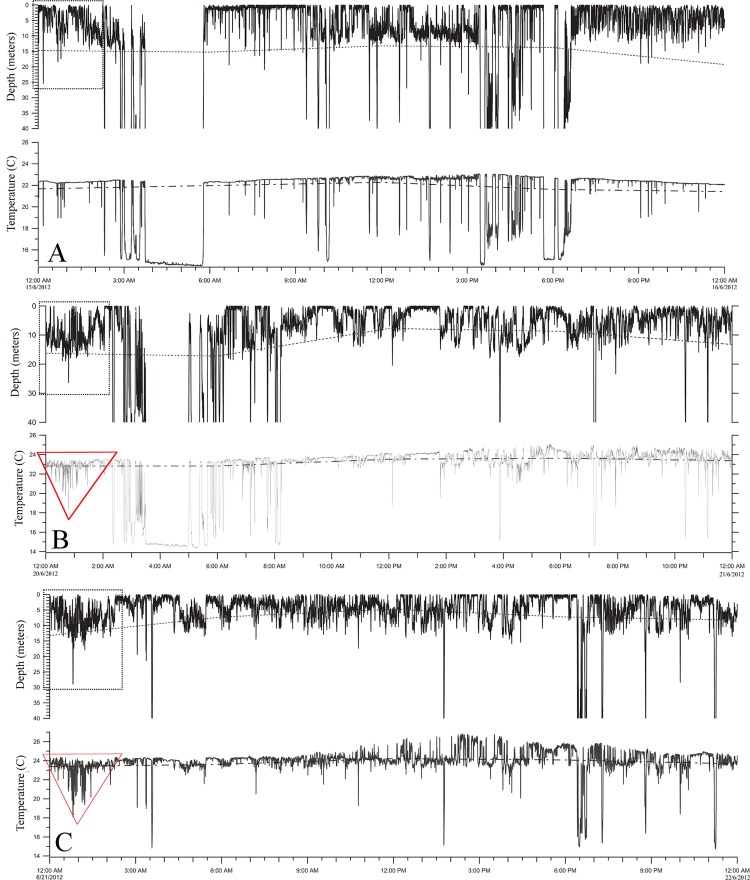
Depth and temperature profiles #10P0648. Dot line corresponds to mixed layer while a dashed line is the thermocline. (A) Cluster 2, the high oscillatory dives are present but do not break the mixed layer (16 June). (B) Cluster 2, high oscillatory dives going below the thermocline, followed by a “U”-shape dive (21 June). (C) Cluster 3 (consecutive day), same pattern but only a few “V”-shape dives during the rest of the day (22 June).

Clusters 2 and 3 for May, June and July were analyzed using Kernel density distribution showing hot-spot areas overlapping with low LI scores (Fig. 10B). These two clusters showed a distinctive diving pattern associated with remarkable use of the thermocline (Fig. 11) that was found only in June. This high oscillation dive patterns, comprised vertical movements between 0 and 30 m, dive signatures crossing the mixed layer are unique to these clusters. Depth increased in the middle of the event period and, as a result of these dives, a thermal footprint in the external temperature profile was left (normally as an inverted triangle Figs. 11A and B). The thermal difference between the highest and the lowest temperature was between 2 and in some cases 6°C and these were detected only at night, from 11:15 pm to 2:30 am with duration between 60 to 120 min. This night diving behavior started on the 7^th^ of June and lasted till the 3^rd^ of July, but the thermocline was only used as described here in the period from the 11^th^ to the 26^th^ of June. For at least 3 days within this last mentioned period the tuna were not using the combination of high oscillation dives below the mixed layer (Fig. 11A).

**Fig 12:**
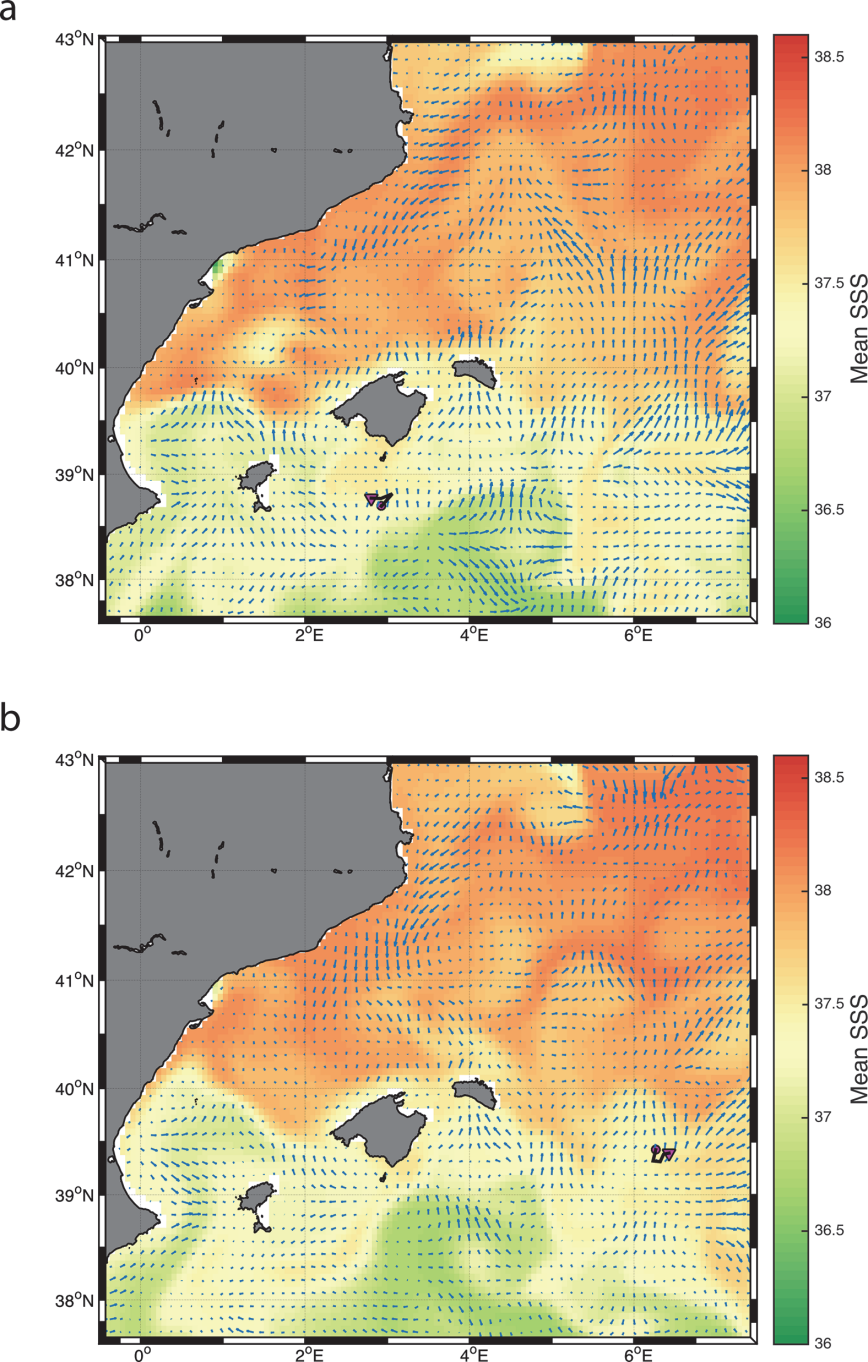
Trajectory of tuna with tag ID # 10P0398 (A) June 16 to June 20 (pink dot and triangle, respectively) and (B) July 6 to July 10 (pink dot and triangle, respectively) plotted with the mean Sea Surface Salinity (SSS) and the mean geostrophic velocity (UV) for those specific days.

The limitation of the data received from the other 3 bluefin tuna forced us to use different approaches to assess their behaviors. The second tuna with tag # 10P0398, which was tagged in Moraira, E Spain on May 29, 2011(43.9 kg and 135 cm) moved to the oceanographic area well known as a region of spawning in the Balearic Islands (Fig. 7). This tuna showed the lowest LI score of all the 4 tuna, indicative of a residency pattern in these waters situated south of Majorca Island, Balearic Islands from the 16^th^ to the 20^th^ of June (LI 0.19). Then the tuna moved eastward and explored a small area between the Balearic Islands and Sardinia, Italy, from the 6^th^ to the 10^th^ of July (LI 0.59, Fig. 8). The mean SST estimated from the tag was 23.15°C (SD ± 0.47) during this June period, while the mean SST estimated in the July period was 24.92°C (SD ± 0.37). Time at temperature for the temperature range 21–24°C was 95.7% (SD ± 2.37) during the June period, and 85.95% (SD ± 14.93) for the temperature range 24–27°C during the July period. This indicates a clear preference for the temperature range 21–27°C, in accordance with what has previously been reported for preferences of tuna presumed spawning in the Balearic Sea [49,51,52]. The bathymetric depth estimated for those locations was 2351.91 m (SD ± 316.29) and 2862.92 m (SD ± 5.06), respectively. Time at depth for the depth range between surface and 20 m for those two periods was 80.16% (SD± 15.93) and 88.14% (SD ± 1.89), revealing a significant surface behavior pattern consistent with what is been reported previously for tunas in spawning areas in the Gulf of Mexico [25,56]. The satellite images for these periods show that the fish was in an area of mixing between Atlantic and Mediterranean waters (Fig. 12). Mean salinity for the first and second period was 37.38 ± 0.07 and 37.47 ± 0.03 respectively (Table 5).

**Table 5:** Satellite oceanographic parameters for the 4 bluefin tuna (BFT) during the proposed spawning periods of tunas with tag ID # 10P0398, 10P0546, 10P0648 and 10P0402.

			CHL-a				NSST				SSS				UV			
Tag ID	Year	Dates	Mean	STD	Min	Max	Mean	STD	Min	Max	Mean	STD	Min	Max	Mean	STD	Min	Max
10P0398	2011	16–20/6	0.0798	0.0120	0.0698	0.0929	22.7407	0.2068	22.5148	22.9121	37.3793	0.0711	37.2828	37.4679	0.1397	0.0492	0.0879	0.1982
	2011	16–20/6	0.0829	0.0010	0.0816	0.0842	23.4162	0.0396	23.3611	23.4672	37.4702	0.0300	37.4327	37.5036	0.1001	0.0370	0.0580	0.1351
10P0402	2012	11–15/6	0.0407	0.0027	0.0384	0.0448	22.7262	0.1714	22.5851	22.9609	38.4328	0.0154	28.4110	38.4534	0.2613	0.0192	0.2373	0.2864
10P0648	2012	26–31/5	0.0869	0.0045	0.0824	0.0939	18.8995	0.2033	18.5897	19.2129	28.7977	0.0970	28.6009	38.8555	0.0488	0.0272	0.0172	0.0920
	2012	11–15/6	0.0687	0.0049	0.0619	0.0751	22.8675	0.0525	22.8031	22.9423	38.9736	0.0236	38.9485	38.9970	0.0995	0.0533	0.0625	0.1914
	2012	21/6 5/7	0.0687	0.0072	0.0588	0.0810	25.6082	1.1883	23.8509	27.4232	39.0079	0.0506	38.8468	39.0622	0.0785	0.0559	0.0248	0.2492
10P0546	2012	26–30/6	0.0884	0.0028	0.0857	0.0919	23.4932	0.1847	23.1804	23.6659	37.9308	0.1451	37.7703	38.0898	0.1955	0.1021	0.0943	0.3658

Average (± SD) and range of Chlorophyll-a (Chl-a), Night Sea Surface Temperature (NSST), Sea Surface Salinity (SSS) and Geostrophic Velocity (UV).

Additional potential spawning fish were identified as tags numbered # 10P0402 and tag # 10P0546. Both bluefin tuna were tagged and released in Roses, NE Spain on August 31 and September 1 2011, respectively. They remained associated to the Gulf of Lions and N and NE Balearic Islands till early May and beginning of June 2012 when they migrated towards the central Mediterranean. For these tags the only data received was light levels and SST. Tuna with tag # 10P0402 started moving towards the central Mediterranean on the 2^nd^ of June and popped-off on the programmed date. Towards the end of its trajectory (from the 26^th^ to the 30^th^ of June 2012), its LI score was 0.55 and at that time the fish was off the northern coast of Tunisia, at the entrance of the Strait of Sicily, after a track of c. 753 km in 28 days (Figs. 7 and 8). The mean SST estimated for this period was 24.02°C (SD ± 0.34), while the estimated mean bathymetry during those days was 369.26 m (SD ± 143.43).

Tag # 10P0546 left the western Mediterranean on the 9^th^ of May and went straight toward the Gulf of Sidra, swimming c.1624 km in 29 days (LI 0.97, SD ± 0.02), where it remained from the 7^th^ to the 20^th^ of June, showing low LI scores from the 11^th^ to 15^th^ and from the 16^th^ to the 20^th^ of June (0.21 and 0.66, respectively (Fig. 8). The mean SST estimated from the tag was 23.35°C (SD ± 0.46) during the first period and 24.22°C (SD ± 0. 45) during the second, with an estimated mean bathymetry of 1048.04 m (SD ± 82.15) and 1199.72 m (SD ± 72.96), respectively. On the 21^st^ of June the tuna left the area towards the W Mediterranean in a straight line (LI 0.99 (SD ± 0.00) till the 30^th^ of June, when its tag popped-off, having swum about 610 km in 9 days (Figs. 7 and 8).

### Satellite Analyses

Satellite oceanographic observations were obtained (i.e. Chl-a, NSST, SSS and UV) for the locations of the bluefin tunas (for tags # 10P0398, 10P0546, 10P0648 and10P0402) during the possible period for potential spawning activity (Table 5). We found that for bluefin tuna # 10P0398 and # 10P0402 all oceanographic parameters examined were within the range of criteria described previously for the Balearic [49,51,52] and Gulf of Mexico [56] spawning grounds. For bluefin tuna # 10P0546 and # 10P0648, whose tracks were in the central Mediterranean, satellite observed values were slightly higher than the theoretical spawning range for Chl-a, NSST and Salinity, described for the western Mediterranean and Gulf of Mexico.

## Discussion

Atlantic bluefin tuna are hypothesized to migrate to the Mediterranean Sea from the Atlantic Ocean during the months of April to May [18] to reproduce, and after spawning in the Mediterranean they have been postulated to immediately initiate a migration out to the Atlantic Ocean to feed [19–21]. Even though this is a well-documented movement pattern in the literature, the results herein reveal residency for long durations and are similar to the recent electronic tagging efforts reported [38,41]. Together the electronic tagging studies indicate that some bluefin tuna are residential within the Mediterranean for much of the year. Of the 39 electronic tags analyzed (including internal tag # 089152), all tagged bluefin tuna remained in the Mediterranean Sea for up to a year post release.

Recent Mediterranean Sea electronic tagging studies, which did not cover the Balearic Sea region, also concluded that after spawning, individuals below 100 kg do not exit the Mediterranean, remaining to forage in areas of high primary production in the Mediterranean Sea. To date, from tagging experiments only those individual tunas over 150 kg demonstrate a pattern of migration into the NE Atlantic Ocean, implying a size dependency to a North Atlantic migration [13,21,43,44]. In this study, although some of the tunas, which were tagged in the northern Balearic Islands, were over 150 kg in size these fish also displayed the residential behaviors typical of the smaller tagged fish. These large bluefin occupied the northern Balearic waters until the thermocline broke down, at which point they started moving away from this region. This residential behavior was also observed for tuna below 100 kg in this study. It has been hypothesized that dispersal movements away from the hot spot region of residency could be related to the drop of water temperatures and the disintegration of the thermocline with increased wind driven mixing around November [73]. Cooling of the water column and loss of the vertical structure may lead to less concentrated prey aggregations which may underlie the movements away from these areas as a reduction in prey availability may force bluefin tuna to move [74].

Two of the tagged tuna, deployed in waters off Roses (NE Spain), visited the Tyrrhenian Sea (bluefin tag # 08A0399 and 0809138). The first tagged fish had its tag pop off along the northern coast of Sicily at the beginning of December, while the second bluefin tuna spent November, December and January there before crossing the Strait of Sicily. Our data also revealed a connection between a tuna tagged in the Adriatic Sea and the western Mediterranean Sea. One individual tuna also showed a lesser known connection between the Adriatic Sea and Libyan waters in winter. In addition, a direct link due to movements between the western Mediterranean Sea population and the Gulf of Sidra waters (Libya) was observed during the spawning season (bluefin tag # 10P0546). Similar types of directed movements were also found by Fromentin & Lopuszanski [41].

Bluefin tunas tagged and released in Corsica, on the other hand, remained highly residential in a reduced area between the waters off Corsica and Sardinia for the entire period of the tracking time recorded (i.e. between 25 and 235 days), potentially suggestive of a forage aggregation for tunas. Moreover, the Algerian basin was also found to be an important foraging area for juvenile tuna. Like all bluefin tuna in the present study, these fish did not cross over to the eastern Mediterranean basin. Rodríguez-Roda [75] reported that a 45 kg tuna which was conventionally tagged in May in Sicily (Italy) appeared off the east Spanish coast in November. Similar results were recently obtained with electronic tagged fish in recently reported work by De la Serna *et al*. [38] who described results from 8 pop up satellite and 2 internal tags deployed in the same area and season as the present study. Moreover, Fromentin & Lopuszanski [41] also deployed 39 pop up satellite tags off Marseille (southern France) in autumn months and they reported an individual (> 150 kg) that visited the Sidra spawning ground at the end of June—in a similar fashion to the tuna with tag # 10P0546 in this study. Similarly, another tuna (approximately 50 kg) whose tag popped off in the Tyrrhenian Sea (north of Sicily) in late April displayed similar behaviors to bluefin tuna with tag # 08A0399 in this study. Aranda *et al*. [36] confirmed the migration of presumably post-spawning adult bluefin tuna from the southern Balearic waters to the Atlantic Ocean after having conducted satellite tagging during the spawning season. Similar to this finding, two individuals (> 150 kg) tagged with peritoneal implantedarchival tags off the eastern coast of USA, exhibited a multi-year site fidelity or homing behaviors to the western Mediterranean basin during the spawning season while overwintering in Atlantic waters [13]. These two archival tagged tunas did not enter the eastern Mediterranean Sea indicative of fidelity to the western Mediterranean known spawning areas. In fact to date, few bluefin tunas reported from the US electronic tagging studies have passed across the central Mediterranean indicative that these western Atlantic tagged bluefin are from populations that originate in the western Mediterranean. Taken together, the results from the previous studies and ours provide a view of the western Mediterranean as an area where bluefin of many year classes are mixing. These bluefin tuna exhibit behaviors suggesting that at least some bluefin adults and sub-adults are residential (current studies), while other fish return to spawn from the North Atlantic foraging grounds. Tagged bluefin show fidelity to the Balearic waters during the spawning season [13], and some post-spawners move out into the Atlantic Ocean after spawning [36] but return again the following spawning year. Although some residency is contributed to the short duration of tag attachments- the fact that longer retention tags also displayed the same behaviors provides further evidence that there are bluefin that are residential throughout the year in the western Mediterranean. Additionally, several recent studies using similar techniques indicate that foraging as well as spawning may be a key driver of retentive behaviors in the Mediterranean for bluefin tuna.

Juvenile bluefin tuna tagged with internal tags that were at large for long durations in the present study (391 and 965 days post release), remained in the western Mediterranean. One internally archival tagged fish displayed a short visit (bluefin tuna tag # 0809138, Fig. 3) to the central Mediterranean basin. Moreover, a long-term aggregation area associated with the Algerian Current system, between the Balearic Islands and the Algerian coast was found for at least one of the archival tagged juveniles (tag # 0809138, Fig. 3). Juvenile bluefin tuna tagged in November in the Adriatic Sea displayed a residential behavior in the central Mediterranean, although one fish moved to the waters south of France after 7 months at liberty and another one visited the Ionian Sea, the Aegean Sea and Libyan waters [35,76]. These results suggest significant residency of juvenile bluefin tuna within the central Mediterranean Sea, consistent with the movements observed for the young adult tuna tagged in the same area in the present study.

Electronic tagging, otolith and genetic results have also shown that significant numbers of juvenile bluefin tuna of Mediterranean origin are migrating into the western Atlantic Ocean to forage [3,13,17,77]. Taken together, the electronic tagging data from all studies indicates there are multiple demographic units of bluefin tuna, mixing in the Mediterranean with distinct behaviors, i.e. some migratory and some resident co- existing together, and mixing within these diverse oceanic regions. The hypothesis that there might be a co-occurrence of residential and migratory bluefin tunas in the Mediterranean has been proposed by many scientists previously (see review in Mather [8]), and even though the demographic relationships between tuna following each pattern at a given time remains unknown, it appears as though electronic tagging is validating the overlap of these behaviorally distinct fish. However, caution must be taken due to the short duration of tracks which do not allow significant time for dispersion and demonstrate the need for longer studies, only achievable with internal tags to garner 1–4 year tracks that may shed light on the population structure. Genetic studies have demonstrated population structuring within the Mediterranean Sea [77]. Combining genetics (identification of population structure with fin clips) and archival tagging would be a way to move forward with resolving the distinctive populations and their migratory trajectories.

Taking into account the weight distribution of bluefin tuna found (approximately 95% of large fish > 100 kg) in the Strait of Gibraltar and Moroccan tuna traps during the spawning migration [42,78,79], and the fact that most of the captures outside the spawning season in different areas of the Mediterranean Sea are small to medium tuna—i.e. eastern Mediterranean [80], Tyrrhenian and Messina Strait [81], South Balearic Islands [82], Strait of Gibraltar [19] and Tunisia [83]-, and the experimental tagging results from various authors [13,26,36,84], we can postulate that part of the Mediterranean highly migratory population comes in as others have suggested from foraging grounds in the north Atlantic. These bluefin tunas, spawned in the Mediterranean Sea, migrate to the North Atlantic to spend their juvenile life phase in the Atlantic Ocean, and after reaching a minimum size of at least 200 cm CFL (i.e. > 140 kg), migrate back into the Mediterranean Sea to spawn. In addition, as demonstrated by Block *et al*. [13], these North Atlantic foragers that spawn in the western Mediterranean, show multiple-year spawning fidelity after they return to the western Mediterranean, with multi-year entrances and exits for up to 4 consecutive years have been recorded on archival tags before their recapture. Block *et al*. [13] documented that, when entering the Mediterranean, these types of Atlantic bluefin tuna are large in body size in comparison to reports of early maturing population of eastern Mediterranean spawners, used in ICCAT assessments [4]. This hypothesis of a highly migratory later maturing bluefin entering the western Mediterranean for spawning is supported by the fact that the documented Mediterranean catches of larger tuna (> 150 kg) only increase during the spawning season [19,80,82,83,85]. Thus, the tagging, genetics and fisheries data taken together indicate that both, residential populations of bluefin, and migratory populations, overlap in the Mediterranean Sea. This should also be of importance to models of bluefin that only use a single age to maturity for Mediterranean bluefin given more than one population exists with distinctly different behaviors. Five of the 39 analyzed tunas in the present study were large fish (> 100 kg), which obviously raises the question about where do they fit in this Mediterranean Sea mosaic. Do these large residential tuna eventually become Atlantic migrants? Or do they always stay part of the resident population? Genetics and long duration tracks from archival or pop up satellite archival tags that are recovered will help establishing which population these particular tagged fish were from. Thus in the future, it is necessary to report a track with a genetic ID so that models incorporating such data know which population of fish they are assigning foraging locations and spawning areas to when modeling what these fish do [5].

Kernel density estimators were successfully used in several tracking studies to describe habitat use and to identify high use areas for marine animals [13,24,26,86,87]. The utilization distributions produced in the present study from the tracking data (Fig. 5) identified overwintering/feeding areas in the waters south of the Gulf of Lions and the Adriatic Sea, primarily the deeper waters of the Jabuka Pit. The Gulf of Lions is known as one of the most productive areas of the Mediterranean Sea due to hydrographic features such as a wide shelf, strong vertical mixing in winter, coastal upwelling and river runoff [88]. The latest sardine and anchovy biomass assessments performed by the General Fisheries Commission for the Mediterranean Sea (GFCM) found that their combined biomass was nearly 1 million tons in the northern Adriatic area [89], accounting as one of the most productive regions in the Mediterranean Sea [90–92]. De la Serna *et al*. [93] analyzed the stomach contents of bluefin tuna in the western Mediterranean and found that the main ingested species were sardine and anchovy (apart from a number of unidentified fish species, i.e. Teleostei spp.). Additionally, and supporting our results, Druon *et al*. [53] detected the Gulf of Lions as potential feeding habitat in a model developed to map potential Atlantic bluefin tuna feeding and spawning habitats in the Mediterranean Sea; while recently, Fromentin & Lopuszanski [41] suggested an overwintering area for adult tuna south of Marseille (France).

Moreover, our results have been found to be in good agreement with the map presenting the overall bluefin tuna fishing operations in the Mediterranean Sea from 1989 to 2012 (Fig. 6). The map provides a comprehensive set of data for fishing locations for the species, thus, it can be considered a robust proxy for tuna aggregations in the Mediterranean, particularly from April to August. Important aggregation areas in the Tyrrhenian Sea and in the eastern Mediterranean, which are evident from the map with the bluefin tuna fishing operation locations (Fig. 6), and are well-known [81,94–98] and references therein, for the Tyrrhenian Sea; and Karakulak and Oray [99] for the eastern Mediterranean—were not visited by the tuna tagged in this study during the spawning season (Fig. 2). No tags were deployed in these two regions, which could have led to these two gaps. Regarding the eastern Mediterranean, Karakulak and Oray [99] stated that a sub-population of bluefin tuna exists in the northern Levantine Sea, which is supported by tagging studies conducted in the eastern Mediterranean where tuna showed a tendency to remain in the Levantine Sea up to 36 days, the furthest one arriving to the Gulf of Laconia (Peloponnese, Greece) after 39 days, and the only fish in the study to visit the central Mediterranean [34].

Histological and larval studies also indicate that this region is a bluefin tuna spawning ground for the eastern Mediterranean basin [99–101]. To date the mixing rates between the eastern, central and western Mediterranean basins have not been adequately resolved. Results from this study and prior works support existence of western Mediterranean bluefin population distinct from an eastern bluefin tuna population.

The Atlantic bluefin tuna population structure within the Mediterranean Sea appears to have a more complex structure than previously thought. Recent genetic studies employing a range of molecular markers have not reached a consensus on the stock structure of bluefin tuna in the Mediterranean [102–110], although more recent genetic studies do agree on the fact that there is evidence of complex population differentiation within the Mediterranean [15–17,111]. Riccioni *et al*. [77] have recently reported structure in relationship to oceanographic conditions providing one hypothesis for how bluefin may be diverging (e.g. preferences for oceanographic areas within the Mediterranean Sea). Advances will only occur when multidisciplinary studies are conducted using all available tools (genetics, tagging and isotopes).

The time series from the archival data of the bluefin tuna # 10P0648 recovered tag allowed us to perform a cluster analysis using custom depth bins to categorize the different diving behavior. Corriero *et al*. [112] found mature tuna of similar size than bluefin tuna # 10P0648 in early July in the north Ionian Sea. Their gonads showed late vitellogenesis with extensive atresia and no post-ovulatory follicles, suggesting that mature females inhabiting non-spawning areas, reabsorb their yolk reserve and do not spawn. These follicles can only be detected for a short time (< 24 h) following spawning in a number of tuna species [113–115], so it can be also interpreted as a sign of cessation of spawning. From the cluster analyses on fish # 10P0648 we concluded that clusters 2 and 3 grouped behavior compatible with previously discussed spawning behaviors observed in the Gulf of Mexico. A high utilization of the surface waters occurred, from 5 to 10 m of the water column. These two clusters were concentrated in May, June and July and the Kernel density analysis for these clusters coincided with low aggregation as represented by LI scores for this tuna. Similar or lower LI were described in adult bluefin tuna spawning in the Gulf of Mexico by Teo *et al*.[56] (i.e LI 0.56 ± 0.1). SST in May registered by the tuna were on average 18.88± 0.83°C and these low temperatures are below the optimal temperature range identified as necessary for spawning, so we discarded this aggregation site and time of year and focused in those areas of low LI scores during June to interpret whether spawning may have occurred using some of the detailed observations of Teo *et al*. [56].

A detailed look at the depth and external temperature archival records of the June days showed that a high oscillation dive behavior appeared around midnight in the archival record. During this period, the fish moved below the mixed layer and we hypothesize that the bluefin tuna potentially uses these movements as a thermoregulatory cooling mechanism. At the thermocline, thermal rate of change is high with small changes of depth (Fig. 11). In most of the cases, after this behavior appeared, and coinciding with the dawn, the tuna made a deep “U”-shaped dive that other authors have interpreted as feeding [24]. The high oscillatory dives combined with the crossing of the mixed layer described above (Results section), occurred only during a 16 day period in June. This behavior was previously described for giant bluefin tuna in the Balearic Islands [36] and has been seen in the Gulf of Mexico (B. Block unpublished data). Moreover, they also found days during the spawning season where this behavior was not detected. Di Natale [54] and Piccinetti [55] suggested that a difference of at least 3°C between the upper and lower stratum of the thermocline is needed as physiological stimulus for spawning; this minimum difference was found in our data during the events described herein.

Bluefin tuna, like other temperate and tropical tunas, are a multiple batch spawner [116–123], due to its asynchronous oocyte development [18,47]. Tuna spawning, therefore, can happen almost daily throughout the reproductive season [113–115,124–126]; in particular the spawning frequency or interval of bluefin tuna in the Mediterranean has been estimated at 1.2 days [18,127]. Bluefin tuna # 10P0648 showed a pattern of residency from the 11^th^ to the 26^th^ of June and simultaneously displayed repetitive vertical behavior during June in the Ionian Sea. The archival record also showed a thermoregulatory behavior that only occurred during this period and not in other areas or times. Potentially this is a behavior indicative of spawning, yet further data are required to confirm such a hypothesis. In both, the Mediterranean and the Gulf of Mexico, the location, size and intensity of spawning can be highly variable among years due to the spatial and temporal variability of the major oceanographic features (e.g. fronts) and environmental conditions (e.g. SST) [3,128]. Bluefin tuna are opportunistic spawners that follow environmental signals and adapt to year-to-year or longer-time variability [50].

The Balearic Sea, and more specifically the ocean area south of the Balearic Archipelago, has been identified as one of the key spawning grounds for Atlantic bluefin tuna within the Mediterranean Sea [3,13,49]. In this study, to investigate when tuna might be spawning in this region, we used both cluster and linearity analyses to discern periods of aggregation with unique behaviors. Considering the LI score, indicative of a sinuous behavior (suggestive of aggregation), bluefin tuna # 10P0398 showed two residential periods, from June 16 to June 20, 2011 (LI 0.19), and from July 6 to July 10, 2011 (LI 0.59). During these two periods the bluefin tuna were occupying the southern waters of the Balearic Islands and the waters between the Balearic Islands and Sardinia, respectively. At that time the fish experienced a period of residency as determined by the linearity index, similar to that observed by electronic tagged bluefin tuna in the Gulf of Mexico spawning season [25].

During both aggregation periods, bluefin tuna # 10P0398 was located in waters of lower salinity (i.e. 37.38 ± 0.07 and 37.47 ± 0.03), indicative of Atlantic and Mediterranean waters mixing, with moderate NSST (i.e. 22.7°C ± 0.2 and 23.4°C ± 0.04) and Chl-a concentrations (0.079 mg/m^3^ ± 0.012 and 0.083 mg/m^3^ ± 0.001) (Table 5). In addition, the timing of the aggregation is in agreement with previous studies where bluefin tuna were found in spawning condition to the south of the Balearic Islands from mid-June to mid-July [2,18,46,47,49,50,78,129]. Frontal structures with mesoscale eddies and anticyclonic gyres have been found to be associated to the presence of bluefin tuna at the time of spawning [49]. These two features, linked to egg and larvae retention, are hypothesized to be utilized by bluefin during the spawning period in the Balearic Archipelago [49,50]. In confirmation of this hypothesis, a high number of bluefin tuna larvae have been found associated with waters of Atlantic origin (i.e. SSS up to 37.2) or with areas where Atlantic and Mediterranean waters mix (i.e. SSS between 37.2 and 38) and with warm waters (over 20.5°C), mostly ranging from 23.5 to 25°C [49,51]. The trajectory from June 16^th^ to June 20^th^ and from July 6 to July 10 of tuna with tag # 10P0398 plotted against the satellite salinity, shows that the fish was in an area where waters of Atlantic origin were mixing with the waters from the Mediterranean Sea. (i.e. SSS of 37.38 ± 0.07 and 37.47 ± 0.03 for the first and second periods, respectively) (Fig. 12 and Table 5).

Larvae of Atlantic bluefin tuna have been found in the Balearic archipelago waters associated to geostrophic velocities ranging from 0.0168 to 0.37 m/s [49], and Chl-a concentrations ranging from 0.08 to 0.15 mg/m^3^ have been found to be preferred by spawning bluefin tuna in the Mediterranean Sea [53]. These values are comparable to the oceanographic conditions we found for tuna with tag # 10P0398 for both periods, i.e. an average UV of 0.139 ± 0.049 m/s and of 0.100 ± 0.037 m/s for the first and second period, respectively; and Chl-a concentrations ranging from 0.069 to 0.092 mg/m^3^ and from 0.082 to 0.084 mg/m^3^ for the first and second period, respectively (Table 5). Alemany *et al*. [49] also found that bluefin tuna showed a preference for deep waters in the Balearic Islands, similar to the deep bathymetric depths at which tuna with tag # 10P0398 was swimming, both, from June 16^th^ to June 20^th^ (i.e. between 2456 and 2588 m) and from July 6 to July 10 (i.e. between 2857 and 2870 m). Moreover, the vertical movements of this bluefin tuna were linked to the surface waters (> 80% of the time spent in the first 20 m), suggested by several authors as a spawning behavior [12,25,36,56]. Due to the lack of an archival record for this tag we cannot postulate that the tuna was spawning but the oceanographic parameters and residential behaviors could be compatible with spawning events at the locations this fish occurred in the region.

The bluefin tuna with tag # 10P0546, also showed behavioral indications of spawning, including a track with a low LI score from June 11^th^ to June 15^th^, 2012. During this period the fish was in the Gulf of Sidra (Libya). The bluefin showed a preference for deep bathymetric depths (i.e. 1048.04 ± 82.15 m), NSST (i.e. mean of 22.74 ± 0.21°C), productivity (i.e. from 0.038 to 0.045 mg/m^3^) and geostrophic velocity (i.e. from 0.237 to 0.286 m/s) (Table 5). Moreover, the fast and long distant movement detected for this tuna between the northwestern Mediterranean and the central Mediterranean (i.e 1624 km in 29 days with high LI scores of 0.97 ± 0.07), with a short period of residency in the Gulf of Sidra, followed by a direct movement with high LI scores (0.99 ± 0.00), suggest a specific purpose compatible with spawning. Hence, this tuna displayed what could be a link between populations in the NW Mediterranean and the Libyan spawning sub-area 4, described by Di Natale [130] and also cited by Fromentin & Lopuszanski [41] in their study for a giant bluefin tuna. Similar results were also found for bluefin tuna with tag # 10P0402, which performed a similar migration towards the central Mediterranean. The pop up satellite archival tag released from this bluefin tuna on the programmed day, preventing us from knowing whether or not the tuna would have remained resident in this area. But the parameters transmitted met the criteria for spawning from June 26^th^ to June 30^th^ (i.e. mean of NSST 23.49 ± 0.18°C, mean of Chl-a 0.088 ± 0.003 mg/m^3^ and mean of SSS 37.93 ± 0.15), in an area off the northern coast of Tunisia, at the entrance of the Strait of Sicily (Fig. 7 and Table 5), which is not a known spawning Mediterranean area.

## Conclusion

The results obtained during the present study provide new information on habitat use of the Mediterranean Sea by sub-adult and adult bluefin tuna. Electronic tagging results indicate extensive residency within the Mediterranean Sea by multiple year classes. The electronic tagging data sets provide the potential to observe residency and behavioral modes and oceanographic conditions associated with potential spawning in the Mediterranean. In this paper we have also identified in addition to known the spawning locations of the western Mediterranean, several potential new spawning areas that may be occurring outside these known areas. In these newly identified regions bluefin displayed behaviors that are consistent with potential spawning behaviors, and the oceanographic features and environmental conditions that bluefin tuna seek for spawning.

Currently used electronic tag technologies provide a choice between implantable archival tags and pop up satellite archival tags. This project occurred during phases of evolving technology. Most of the pop-up satellite tags used in this study were MK10s and large in size. These tags prematurely released, making it challenging to obtain a long duration track. However, the reduction in size of tags, the improved retention with the double attachment, has the potential to increase the days at liberty and length of the data sets acquired. To fully build a complete picture of the bluefin tuna habitat utilization of the Mediterranean and given the mixing evident of populations within the Mediterranean Sea, all future tagging should really be accompanied by other population identification technologies currently available (genetic ID of clip fins, otolith microchemistry and isotopes). Used in combination they offer the potential to finally unravel the mystery of bluefin tuna migrations and utilization of the Mediterranean Sea. The bluefin tuna patterns and population structure need to be understood in order to properly manage and conserve the species and the fishery.

## Supporting Information

S1 Fig Trajectories of all the Adriatic Sea tagged tuna analyzed in this study (Green triangle is deployment position and red triangle is the pop-off position) (PDF)

S2 Fig Trajectories of all the western Mediterranean Sea tagged tuna analyzed in this study (Green triangle is deployment position and red triangle is the pop-off position). (PDF)

S3 Fig Trajectories of all Corsica tagged tuna analyzed in this study (Green triangle is deployment position and red triangle is the pop-off position). (PDF)

S1 Table Average, Standard Deviation, Minimum and Maximum of the daily estimated position variance for the trajectories of the four tunas that showed possible reproductive behavior in the present study. (DOCX)

## References

[1] Cort L, Deguara S, Galaz T, Mèlich B, Artetxe I, et al (2013) Determination of L max for Atlantic Bluefin Tuna, *Thunnus thynnus* (L.), from meta-analysis of published and available biometric data. Fish Sci 21:2, 181–212

[2] Fromentin JM, Powers JE (2005) Atlantic bluefin tuna: Population dynamics, ecology, fisheries and management. Fish Fish 6: 281–306.

[3] Rocker JR, Alvaro-Bremer J, Block BA, De Metrio G, Corriero A, et al (2007) Life story and stock structure of Atlantic Bluefin tuna (*Thunnus thynnus*). Review in Fish Sci 15:265–310.

[4] Anon. (2009) Report of the 2008 Atlantic bluefin tuna stock assessment session. Col Vol Sci Pap ICCAT 64(1): 1–352.

[5] Taylor NG, McAllister MK, Lawson GL, Carruthers T, Block BA (2011) Atlantic Bluefin Tuna: A novel multistock spatial model for assessing population biomass. PLoS ONE 6 (12): e27693 10.1371/journal.pone.0027693 22174745 PMC3235089

[6] Anon. (2007) Report of the 2006 Atlantic bluefin tuna stock assessment session. Col Vol Sci Pap ICCAT 60 (3): 652–880.

[7] MacKenzie BR, Mosegaard H, Rosenberg AA (2009) Impending collapse of bluefin tuna in the northeast Atlantic and Mediterranean. Cons Lett 2 (1): 26–35.

[8] Mather FJ III, JM Mason Jr, Jones AC (1995) Historical document: Life history and fisheries of Atlantic bluefin tuna. NOAA Technical Memorandum NMFS-SEFSC-370.

[9] McGowen MF, Richards WJ (1986) Distribution and abundance of bluefin tuna (*Thunnus thynnus*) larvae in the Gulf of Mexico in 1982 and 1983 with estimates of the biomass and population size of the spawning stock for 1977. 1978, and 1981–1983. Int Comm Conserv Atl Tunas Coll Vol Sci Pap Madrid 24 (2):182–195.

[10] Block BA, Dewar H, Farwell CJ, Prince ED (1998) A new satellite technology for tracking the movements of Atlantic bluefin tuna. Proc Natl Acad Sci USA 95(9384–9389). 9689089 10.1073/pnas.95.16.9384 PMC21347

[11] Lutcavage ME, Brill RW, Skomal GB, Chase BC, Howey PW (1999) Results of pop-up satellite tagging of spawning size class fish in the Gulf of Maine: do North Atlantic bluefin tuna spawn in the mid-Atlantic? Can J Fish Aquat Sci 56: 173–177.

[12] Block BA, Dewar H, Blackwell SB, Williams TD, Prince ED, et al (2001) Migratory movements, depth preferences, and thermal biology of Atlantic bluefin tuna. Science 293 (5533): 1310–1314. 11509729 10.1126/science.1061197

[13] Block BA, Teo SLH, Walli A, Boustany A, Stokesbury MJW, et al (2005) Electronic tagging and population structure of Atlantic bluefin tuna. Nature 434: 1121–1127. 15858572 10.1038/nature03463

[14] Rooker JR, Secor DH, De Metrio G, Schloesser R, Block BA, Neilson JD (2008) Natal homing and connectivity in Atlantic bluefin tuna populations. Science 322: 742–744. 10.1126/science.1161473 18832611

[15] Carlsson J, McDowell JR, Carlsson JEL, Graves JE (2007) Genetic Identity of YOY Bluefin tuna from the eastern and western Atlantic spawning areas. J Hered 98 (1): 23–28. 17158466 10.1093/jhered/esl046

[16] Boustany A, Reeb CA, Block BA (2008) Mitochondrial DNA and electronic tracking reveal population structure of Atlantic bluefin tuna (*Thunnus thynnus*). Mar Biol 156: 13–24.

[17] Riccioni G, Landi M, Ferrara G, Milano I, Cariani A, et al (2010) Spatio-temporal population structuring and genetic diversity retention in depleted Atlantic Bluefin tuna of the Mediterranean Sea. Proc Natl Acad Sci USA 107 (5): 2102–2107. 10.1073/pnas.0908281107 20080643 PMC2836650

[18] Medina A, Abascal FJ, Megina C, García A (2002) Stereological assessment of the reproductive status of female Atlantic northern bluefin tuna during migration to Mediterranean spawning grounds through the Strait of Gibraltar. J Fish Biol 60: 203–217.

[19] Serna JM, Alot E, Majuelos E, Rioja P (2004) La migración trófica post-reproductiva del atún rojo (*Thunnus thynnus*) a través del Estrecho de Gibraltar. Col Vol Sci Pap ICCAT 56 (3):1196–2109.

[20] De Metrio G, Arnold GP, de la Serna JM, Block BA, Megalofonou P, et al (2005) Movements of bluefin tuna (*Thunnus thynnus* L.) tagged in the Mediterranean Sea with pop-up satellite tags. Col Vol Sci Pap ICCAT 58 (4): 1337–1340.

[21] De Metrio G, Arnold GP, de la Serna JM, Megalofonou P, Sylos Labini G, et al. (2005) Movements and migrations of North Atlantic Bluefin tuna tagged with pop-up satellite tags. Aquatic Telemetry: advances and applications. Proceedings of the 5^th^ Conference on Fish Telemetry held in Europe, Ustica, Italy, FAO/COISPA, Rome.

[22] Lutcavage ME, Brill RW, Skomal GB, Chase GJL, Tutein J (2000) Tracking adult North Atlantic bluefin tuna (*Thunnus thynnus*) in the northwestern Atlantic using ultrasonic telemetry. Mar Biol 137: 347–358.

[23] Stokesbury MJW, Teo SLH, Seitz A, O′Dor RK, Block BA (2004) Movement of Atlantic bluefin tuna (*Thunnus thynnus*) as determined by satellite tagging experiments initiated off New England. Can J Fish Aquat Sci 61: 1976–1987.

[24] Wilson SG, Lutcavage ME, Brill RW, Genovese MP, Cooper AB, Everly AW (2005) Movements of bluefin tuna (*Thunnus thynnus*) in the northwestern Atlantic Ocean recorded by pop-up satellite archival tags. Mar Biol 146: 409–423.

[25] Teo SLH, Boustany A, Block BA (2007) Oceanographic preferences of Atlantic bluefin tuna, *Thunnus thynnus*, on their Gulf of Mexico breeding grounds. Mar Biol 152: 1105–1119.

[26] Walli A, Teo SLH, Boustany A, Farwell CJ, Williams T, et al (2009) Seasonal movements, aggregations and diving Behavior of Atlantic Bluefin Tuna (*Thunnus thynnus*) Revealed with archival tags. PLoS ONE 4 (7): e6151 10.1371/journal.pone.0006151 19582150 PMC2701635

[27] Lawson GL, Castleton MR, Block BA (2010) Movements and diving behavior of Atlantic bluefin tuna *Thunnus thynnus* in relation to water column structure in the northwestern Atlantic. Mar Ecol Prog Ser 400: 245–265.

[28] Galuardi B, Royer F, Golet W, Logan J, Neilson J, et al (2010) Complex migration routes of Atlantic bluefin tuna (*Thynnus thynnus*) question current population structure paradigm. Can J Fish Aquat Sci 67: 966–976.

[29] Galuardi B, Lutcavage M (2012) Dispersal routes and habitat utilization of juvenile Atlantic bluefin tuna, *Thunnus thynnus*, tracked with Mini PSAT and archival tags. PLoS ONE 7 (5): e37829 10.1371/journal.pone.0037829 22629461 PMC3358288

[30] De la Serna JM, Alot E, Godoy MD (1992) Resultados de la campaña de marcado de atún rojo (*Thunnus thynnus*) realizada en el Mediterraneo Occidental en el año 1990. Condiciones ambientales observadas. Col Vol Sci Pap ICCAT 39: 692–699.

[31] De la Serna JM, Alot E (1993) Resultados de la campaña de marcado de atún rojo (*Thunnus thynnus*) realizada en el Mediterráneo Occidental en el año 1991. Col Vol Sci Pap ICCAT 40: 116–118.

[32] Relini M, Polandri G, Torchia G (1995) Tagging of *Thunnus thynnus* juveniles in the Ligurian Sea, Autumn 1994. Col Vol Sci Pap ICCAT 44: 378.

[33] Arregui I, Arrizabalaga H, de la Serna JM (2006) Preliminary approach to the experimental design of tagging campaigns for movement rates estimation of East Atlantic bluefin tuna. Col Vol Sci Pap ICCAT 59 (3): 769–788.

[34] De Metrio G, Oray I, Arnold GP, Lutcavage ME, Deflorio M, et al (2004) Joint Turkish-Italian research in the Eastern Mediterranean: bluefin tuna tagging with pop-up satellite tags. Col Vol Sci Pap ICCAT 56 (3): 1163–1167.

[35] Yamashita H, Miyabe N (2001) Report of bluefin tuna archival tagging conducted by Japan in 1999 in the Adriatic Sea. Col Vol Sci Pap ICCAT 52 (2): 809–823.

[36] Aranda G, Abascal FJ, Varela JL, Medina A (2013) Spawning behaviour and post-spawning migration patterns of Atlantic Bluefin tuna (*Thunnus thynnus*) ascertained from satellite archival tags. PLoS ONE 8(10): e76445 10.1371/journal.pone.0076445 24098502 PMC3788109

[37] Fromentin JM (2009) Tagging bluefin tuna in the Mediterranean Sea: Challenge or Mission: Impossible? Col Vol Sci Pap ICCAT 65(3): 812–821.

[38] De la Serna JM, Abascal FJ, Godoy MD (2011) Resultados preliminares de las actividades de marcado de atún rojo (*Thunnus thynnus*) realizadas por la Confederación Española de Pesca marítima Recreativa Responsable (CEPRR) con la coordinación científica del Instituto Español de Oceanografía (IEO). Col Vol Sci Pap ICCAT 66 (2): 984–988

[39] Tudela S, Sainz-Trápaga S, Cermeño P, Hidas E, Graupera E, Quílez-Badia G (2011) Bluefin tuna migratory behavior in the western and central Mediterranean Sea revealed by electronic tags. Col Vol Sci Pap ICCAT 66 (3): 1157–1169.

[40] Cermeño P, Tudela S, Quílez-Badia G, Sainz-Trápaga S, Graupera E (2012) New data on buefin tuna migratory behavior in the western and central Mediterranean Sea. Col Vol Sci Pap ICCAT 68 (1): 151–162.

[41] Fromentin JM, Lopuszanski D (2013) Migration, residency, and homing of bluefin tuna in the western Mediterranean Sea. ICES J Mar Sci doi:10.1093/icesjms/fst157.

[42] Quílez-Badia G, Cermeño P, Tudela S, Sainz-Trápaga S, Graupera E (2013) Spatial movements of bluefin tuna revealed by electronic tagging in the Mediterranean Sea and in the Atlantic waters of Morocco in 2011. Col Vol Sci Pap ICCAT 69 (1): 435–453.

[43] Sarà R (1964) Data observations and comments on the occurrence, behaviour, characteristics and migrations of tunas in the Mediterranean. Proc Tech Pap Gen Fish Counc Medit 7: 371–388.

[44] Sarà R (1973) Sulla biología dei tonni (*Thunnus thynnus* L.) modelli di migrazione e di comportamento. Bolletino di Pesca, Piscicultura e Hidrobiología, Roma 28: 217–243.

[45] Susca V, Corriero A, Bridges CR, De Metrio G (2001) Study of the sexual maturity of female bluefin tuna: Purification and partial characterization of vitellogenin and its use in an enzyme-linked immunosorbent assay. J Fish Biol 58: 815–831.

[46] Sarasquete C, Cardenas S, De Canales MLG, Pascual E (2002) Oogenesis in the bluefin tuna, *Thunnus thynnus* L.: A histological and histochemical study. Histol Histopathol 17: 775–788. 12168787 10.14670/HH-17.775

[47] Corriero A, Desantis S, Deflorio M, Acone F, Bridges CR et al (2003) Histological investigation on the ovarian cycle of the bluefin tuna in the western and central Mediterranean. J Fish Biol 63: 108–119.

[48] Platonenko S, de la Serna JM (1997) Observaciones oceanográficas y medioambientales en el Mediterráneo occidental durante la época de reproducción del atún rojo (*Thunnus thynnus* L. 1758). Col Vol Sci Pap ICCAT 46 (4): 496–501.

[49] Alemany F, Quintanilla L, Velez-Belchí P, García A, Cortés D, et al (2010) Characterization of the spawning habitat of Atlantic bluefin tuna and related species in the Balearic Sea (western Mediterranean). Progr Oceanogr 86 (1–2): 21–38

[50] Reglero P, Ciannelli L, Alvarez-Berastegui D, Balbín R, López-Jurado JL, Alemany F (2012) Geographically and environmentally driven spawning distributions of tuna species in the western Mediterranean Sea. Mar Ecol Prog Ser 463: 273–284.

[51] García A, Alemany F, Velez-Belchi P, Lopez Jurado JL, Cortes D, et al (2005) Characterization of the bluefin tuna spawning habitat off the Balearic Archipelago in relation to key hydrographic features and associated environmental conditions. Col Vol Sci Pap ICCAT 58: 535–549.

[52] García A, Alemany F, de la Serna JM, Oray I, Karakulak S, et al (2005) Preliminary results of the 2004 bluefin tuna larval surveys off different Mediterranean sites (Balearic Archipelago, Levantine Sea and the Sicilian Channel). Col Vol Sci Pap ICCAT 58 (4): 1420–1428.

[53] Druon N, Fromentin JM, Aulanier F, Heikkonen J (2011) Potential feeding and spawning habitats of Atlantic bluefin tuna in the Mediterranean Sea. Mar Ecol Prog Ser 439: 223–240.

[54] Di Natale A (2010) The eastern Atlantic bluefin tuna: Entrangled in a big mess, possibility far from a conservation red alert. Some comments after the proporsal to include bluefin tuna in CITES Appendix I. Col Vol Sci Pap ICCAT 65 (3): 1004–1043.

[55] Piccinetti C, Di Natale A, Arena P (2012) Eastern bluefin tuna (*Thunnus thynnus*, L.) reproduction and reproductive areas and seasons. Col Vol Sci Pap ICCAT, 69 (2): 891–912.

[56] Teo SLH, Boustany A, Dewar H, Stokesbury MJW, Weng KC et al (2007) Annual migrations, diving behavior, and thermal biology of Atlantic bluefin tuna, *Thunnus thynnus*, on their Gulf of Mexico breeding grounds. Mar Biol 151: 1–18.

[57] Parrack ML, Phares PL (1979) Aspects of growth of Atlantic bluefin tuna determined from mark-recapture data. Col. Vol. Sci. Pap. ICCAT 8(2): 356–366.

[58] Arena P (2006). Length-weight relationships adopted by the SCRS for major species. Available: http://iccat.int/Documents/SCRS/Manual/Appendices/Appendix%204%20III%20Length-weight.pdf. Accessed 2015 January 13.

[59] Boustany AM, Matteson R, Castleton M, Farwell C Block BA (2010) Movements of pacific bluefin tuna (Thunnus orientalis) in the Eastern North Pacific revealed with archival tags. Prog in Oceanogr 86:94–104

[60] Sibert J, Musyl MK, Brill RW (2003) Horizontal movements of bigeye tuna (*Thunnus obesus*) near Hawaii determined by Kalman filter analysis of archival tagging data. Fish Oceanog 12: 141–151.

[61] Royer F, Fromentin JM, Gaspar P (2005) A state-space model to derive bluefin tuna movement and habitat from archival tags. Oikos 109: 473–484.

[62] Nielsen A, Bigelow KA, Musyl MK Sibert JR (2006) Improving light-based geolocation by including sea surface temperature. Fish Oceanogr 14: 314–325.

[63] Nielsen A, Sibert JR (2007) State-space model for light-based tracking of marine animals. Can J Fish Aquat Sci 64: 1055–1068.

[64] Teo SLH, Boustany A, Blackwell S, Walli A, Weng KC, Block BA (2004) Validation of geolocation estimates based on light level and sea surface temperature from electronic tags. Mar Ecol Prog.Ser 283, 81–98 (2004).

[65] Block BA, Jonsen ID, Jorgensen SJ, Winship AJ, Shaffer SA, et al. (2011) Tracking apex marine predator movements in a dynamic ocean. Nature, DOI:10.1038/nature10082 10.1038/nature10082 21697831

[66] FAO (2013) CWP Handbook of Fishery Statistical Standards. Section H: Fishing areas for statistical purposes. 1. FAO Major Fishing Areas. Available: http://www.fao.org/fishery/cwp/handbook/H/en. Accessed 2015 January 13.

[67] Spencer SR, Cameron GN, Swihart RK (1990) Operationally defining home range: temporal dependence exhibited by hispid cotton rats. Ecol 71:1817–1822

[68] Jorgensen SJ, Arnoldi NS, Estess EE, Chapple TK, Rückert M, et al (2012) Eating or meeting? Cluster analysis reveals intricacies of white shark (*Carcharodon carcharias*) migration and offshore behavior. PLoS ONE 7(10): e47819 10.1371/journal.pone.0047819 23144707 PMC3483152

[69] Abdelhadi B, Hernández Hernández P, Forcada A(2011) Étude de la distribution spatio-temporelle de la pêcherie du thon rouge (*Thunnus thynnus*) en Algérie avec l’utilisation du système d’information géographique (SIG). Mediterránea. Serie de Estudios Biológicos. Época II. N. especial.

[70] Bahurel P, Adragna F, Bell MJ, Jacq F, Johannessen JA, et al. (2009. Ocean monitoring and forecasting core services: The European MyOcean example. OceanObs ’09 OceaInformation for Society: Sustaining the Benefits Realizing the Potential. September 21–25, 2009, Venice, Italy.

[71] van Winkle W (1975) Comparison of several probabilistic home-range models. J Wild Manage 39: 118–123.

[72] R_Development_Core_Team (2013) R: A language and environment for statistical vomputing R Foundation for statistical computing, Vienna, Austria Available: http://www.R-project.org/. Accessed 2015 January 13. 10.1007/s13197-013-0993-z

[73] Salat J, Tudela S, Sainz-Trapaga S, Cermeño P, Quilez-Badia G (2011) Are bluefin tuna fishes in the Mediterranean waiting for a sign from sky to start their migrations? 13^th^ Plinius Conference on Mediterranean Storms. Savona, Italy, 07–09 September 2011, Vol. 13, Plinius13–87, 2011.

[74] Würtz M (2010) Mediterranean pelagic habitat: Oceanographic and biological processes, An overview IUCN, Gland, Switzerland and Malaga, Spain

[75] Rodríguez-Roda J (1969) El atún, *Thunnus thynnus* (L.), del sur de España en la campaña almadrabera del año 1967, y estudio de la evolución de la pesquería de Barbate. Inv Pesq 33 (1): 87–96.

[76] FAO (2006) FAO General Fisheries Commission for the Mediterranean/Commission générale des pêches pour la Méditerranée. Report of the eighth session of the Scientific Advisory Committee. Tirana, Albania, 25–28 October 2005/Rapport de la huitième session du Comité scientifique consultatif. Tirana, Albanie, 25–28 octobre 2005. *FAO Fisheries Report/FAO Rapport sur les pêches*. No. 789. FAO, Rome, 96p.

[77] Riccioni G, Stagioni M, Landi M, Ferrara G, Barbujani G, et al (2013) Genetic structure of Bluefin tuna in the Mediterranean Sea correlates with environmental variables. PLoS ONE 8(11): e80105. doi:. 10.1371/journal.pone.0080105 24260341 PMC3832436

[78] Heinisch G, Corriero A, Medina A, Abascal FJ, de la Serna JM, et al (2008) Spatial–temporal pattern of blueWn tuna (*Thunnus thynnus* L. 1758) gonad maturation across the Mediterranean Sea. Mar Biol 154: 623–630.

[79] Idrissi M, Abid N (2010) Preliminary analysis of the size data of bluefin tuna (*Thunnus thynnus*) caught by the Moroccan Atlantic traps during 2009. Col. Vol Sci Pap ICCAT 65(3): 968–974.

[80] Oray I, Karakulak FS (1997) Some remarks on the bluefin tuna (*Thunnus thynnus* L. 1758) fishery in Turkish waters in 1993, 1994, 1995. Col Vol Sci Pap ICCAT 46 (2): 357 362.

[81] Di Natale A, Mangano A, Asaro A, Bascone B, Celona A, Valastro M (2005) Bluefin tuna (*Thunnus thynnus* L.) catch composition in the Tyrrhenian Sea and in the Straits of Sicily in 2002 and 2003. Col Vol Sci Pap ICCAT 58 (4): 1296–1336.

[82] Fromentin JM (2004) The 2002 size composition of bluefin tuna catches of the French purse seine compared to those of the early 1990s and 2001. Col Vol Sci Pap ICCAT 56 (3):1182–1188.

[83] Hattour A (2003) La peche du thon rouge a la senne tournante en Tunisie: Annee 2001. Col Vol Sci Pap ICCAT 55 (1):204–216.

[84] Stokesbury MJW, Cosgrove R, Boustany A, Browne D, Teo SLH, et al (2007) Results of satellite tagging on Atlantic bluefin tuna, *Thunnus thynnus*, off the coast of Ireland. Hydrobiologia 582: 91–97

[85] Di Natale A (2005) Bluefin tuna (*Thunnus thynnus* L) line fisheries in the Italian Seas. Old and recent data. Col Vol Sci Pap ICCAT 58 (4):1285–1295 (2–5)

[86] Peckham SH, Diaz DM, Walli A, Ruiz G, Crowder LB, et al (2007) Small-scale fisheries bycatch jeopardizes endangered Pacific loggerhead turtles. PLoS ONE 2(10): e1041 17940605 10.1371/journal.pone.0001041 PMC2002513

[87] Graham RT, Witt MJ, Castellanos DW, Remolina F, Maxwell S, et al (2012) Satellite tracking of manta rays highlights challenges to their conservation. PLoS ONE 7(5): e36834 10.1371/journal.pone.0036834 22590622 PMC3349638

[88] Lloret J, Lleonart J, Solé I (2000) Time series modelling of landings in Northwest Mediterranean Sea. ICES J Mar Sci 57: 171–184. doi:10.1006/jmsc.2000.057

[89] FAO (2013) FAO General Fisheries Commission for the Mediterranean/Commission générale des pêches pour la Méditerranée. Report of the fifteenth session of the Scientific Advisory Committee. Rome, 8–11 april 2013/Rapport de la quinzième session du Comité scientifique consultatif. Rome, 8–11 avril 2013. FAO Fisheries and Aquaculture Report/FAO Rapport sur les pêches et l’aquaculture. No. 1042. FAO, Rome, 96p.

[90] Buljan M (1964) Estimation of productivity of the Adriatic based on its hydrographic properties. Acta Adriat 11: 35–45.

[91] Sournia A (1973) La production primaire planctonique en Méditerranée. Essai de mise à jour. Bulletin Etude en commun de la Méditerranée 5: 1–128.

[92] Morovic M, Grbec B, Marasovic I (2004) Changed patterns of remotely sensed chlorophyll a in the Adriatic—Influence of meteorological conditions. Gayana (Concepción) 68: 405–410.

[93] De la Serna JM (2012) Preliminary studies on the feeding of bluefin tuna (*Thunnus thynnus*) in the Mediterranean and the Strait of Gibraltar area. Col Vol Sci Pap ICCAT 68(1): 115–132.

[94] Arena P (1963) Observations dans la partie sud de la mer Tyrrhénienne sur les habitudes et le comportement du thon rouge (*Thunnus thynnus*, L.) pendant sa période génétique. FAO Proc Gen Fish Coun Médit 7: 395–411.

[95] Arena P (1981) Osservazioni sulle concentrazioni e sulla pesca del Tonno e dell’Alalunga nelle zone di mare meridionali. Quad Lab Tecn Pesca 3 (1 suppl.): 77–79.

[96] Arena P (1982) Biologia, ecologia e pesca del tonno, *Thunnus thynnus* (L.), osservati in un quinquennio nel Tirreno meridionale. Atti Conv UU.OO: sottop Ris Biol Inq Marino Roma: 361–405.

[97] Arena P, (1988) Risultati delle osservazioni sulle affluenze del tonno nel Tirreno e sull’andamento della pesca da parte delle tonnare volanti nel triennio 1984–1986. MMM-CNR, Atti Seminari UU.OO. Resp Prog Ric. Roma: 273–300.

[98] Di Natale A, Mangano A, Asaro A, Bascone B, Celona A, et al (2006) Bluefin tuna (*Thunnus thynnus* L.) catch composition in Tyrrhenian and in Sicily Strait in 2004. Col Vol Sci Pap ICCAT, 59 (3): 829–842.

[99] Karakulak FS, Oray I (2009) Remarks on the fluctuations of bluefin tuna catches in Turkish waters. Col. Vol Sci Pap ICCAT 63: 153–160.

[100] Karakulak FS, Oray I, Corriero A, Deflorio M, Santamaria, et al (2004) Evidence of a spawning area for the bluefin tuna (*Thunnus thynnus* L.) in the eastern Mediterranean. J Appl Ichthyol 20: 318–320.

[101] Oray I, Karakulak FS, Alicli Z, Ates C, Kahraman A (2005) First evidence of spawning in the Eastern Mediterranean Sea—Preliminary results of tuna larval survey in 2004. Col Vol Sci Pap ICCAT 58 (4): 1341–1347.

[102] Edmunds PH, Sammons JI (1973) Similarity of genetic polymorphism of tetrazolium oxidase in bluefin tuna (*Thunnus thynnus*) from the Atlantic coast of France and the western North Atlantic. J Fish Res Board Can 30: 1031–1032.

[103] Thompson HC Jr, Contin RF (1980) Electrophoretic study of Atlantic bluefin tuna (*Thunnus thynnus*) from the eastern and western north Atlantic Ocean. Col Vol Sci Pap ICCAT 9 (2): 499–505.

[104] Broughton RE, Gold JR (1997) Microsatellite development and survey of variation in northern bluefin tuna (*Thunnus thynnus)* . Mol Mar Biol Biotechnol 6: 308–314.

[105] Alvarado Bremer JR, Naseri I Ely B (1999) A Provisional Study of Northern Bluefin Tuna Populations. Col Vol Sci Pap ICCAT 49 (1): 127–129.

[106] Takagi M, Okamura T, Chow S, Taniguchi N (1999) PCR primers for microsatellite loci in tuna species of the genus *Thunnus* and its application for population genetic study. Fish Sci 65: 571–576.

[107] Ely B, Stoner DS, Alvarado Bremer JR, Dean JM, Addis P, et al (2002) Analyses of nuclear ldhA gene and mtDNA control region sequences of Atlantic Northern bluefin tuna populations. Mar Biotechnol 4: 583–588. 14961233 10.1007/s10126-002-0040-y

[108] Pujolar JM, Roldán MI, Pla C (2003) Genetic analysis of tuna populations, *Thunnus thynnus thynnus* and *T*. *alalunga* . Mar Biol 143: 613–621.

[109] Alvarado Bremer JR, Viñas J, Mejuto J, Ely B, Pla C (2005) Comparative phylogeography of Atlantic bluefin tuna and swordfish: the combined effect of vicariance, secondary contact, introgression, and population expansion on the regional phylogenies of two highly migratory pelagic fishes. Mol Phylogenet Evol 36: 169–187. 15904864 10.1016/j.ympev.2004.12.011

[110] Carlsson J, McDowell JR, Diaz-Jaimes P, Carlsson JEL, Boles SB, et al (2004) Microsatellite and mitochondrial DNA analyses of Atlantic bluefin tuna (*Thunnus thynnus thynnus*) population structure in the Mediterranean Sea. Mol Ecol 13, 3345–3356. 15487994 10.1111/j.1365-294X.2004.02336.x

[111] Cannas R, Ferrara G, Milano I, Landi M, Cariani, et al (2012) Spatio-temporal genetic variation of Atlantic bluefin tuna from Sardinian and Mediterranean tuna traps. Col Vol Sci Pap ICCAT 67 (1): 351–358.

[112] Corriero A, Karakulak S, Santamaria N, Deflorio M, Spedicato D, et al (2005) Size and age at sexual madurity of female bluefin tuna (*Thunnus thynnus* L. 1758) from the Mediterranean Sea. J Appl Ichthyol 21, 483–486.

[113] Hunter JR, Lo NCH, Leong RJH (1985) Batch fecundity in multiple spawning species. In: An Egg Production Method for Estimating Spawning Biomass of Pelagic Fish: Application to the Northern Anchovy, *Engraulis mordax*. R. Lasker (Ed.). U.S. Department of Commerce, NOAA Technical Report NMFS, 36: 67–77.

[114] McPherson GR (1991) Reproductive biology of yellowfin tuna in the eastern Australian fishing zone, with special reference to the north-western Coral Sea. Aust J of Mar and Freshw Res 42: 465–477.

[115] Schaefer KM (1996) Spawning time, frequency, and batch fecundity of yellowfin tuna, *Thunnus albacares*, near Clipperton Atoll in the eastern Pacific Ocean. Fish Bull 94: 98–112.

[116] June FC (1953) Spawning of yellowfin tuna in Hawaiian waters. U. S. Fish and Wildlife Service, Fish. Bull. 54: 47−64.

[117] Yuen, HSH (1955) Maturity and fecundity of bigeye tuna in the Pacific. U.S. Fish and Wildlife Service Special Scientific Report: Fisheries 150. Washington, D.C., 30 p.

[118] Buñag DM (1956) Spawning habits of some Philippine tuna based on diameter measurements of the ovarian ova. Philipp. J. Fish. 26: 877−920.

[119] Otsu T, Uchida RN (1959) Sexual maturity and spawning of albacore in the Pacific Ocean. U. S. Fish and Wildlife Service, Fish Bull 59: 287−305.

[120] Wallace RA, Selman K (1981) Cellular and dynamic aspects of oocyte growth in teleosts. Am Zool 21: 325–343.

[121] Baglin RE Jr (1982) Reproductive biology of western Atlantic bluefin tuna. Fish Bull 80: 121–134.

[122] de Vlaming VL (1983) Oocyte development patterns and hormonal involvements among teleosts In: Control Processes in Fish Physiology. Rankin J. C., Pitcher T. J. and Duggan R. (Ed.). Wiley-Interscience, New York 176–199.C.

[123] Stéquert B, Ramcharrun B (1995) La fécondité du listao (*Katsuwonus pelamis*) de l’ouest de l’océan Indien. Aquat Living Resour 8: 79–89.

[124] Farley JH Davis TKI (1998) Reproductive dynamics of southern bluefin tuna, *Thunnus maccoyii* . Fish Bull 96: 223–236.

[125] Schaefer KM (1998) Reproductive biology of yellowfin tuna (*Thunnus albacares*) in the eastern Pacific Ocean. Inter-Am Trop Tuna Comm Bull 21(5): 205–221.

[126] Schaefer KM (2001) Reproductive biology of tunas In: Fish Physiology. Block BA, Stevens E (Ed.). Academic Press, San Diego,CA, Volume 19 Tuna: Physiology, Ecology, and Evolution 225–270.

[127] Aranda G, Medina A, Santos A, Abascal FJ, Galaz T (2013) Evaluation of Atlantic bluefin tuna reproductive potential in the western Mediterranean Sea. J Sea Res 76: 154–160.

[128] Muhling BA, Lamkin JT, Roffer MA (2010) Predicting the occurrence of Atlantic bluefin tuna (*Thunnus thynnus*) larvae in the northern Gulf of Mexico: building a classification model from archival data. Fish Oceanogr 19(6): 526–539.

[129] Abascal FJ, Megina C, Medina A (2004) Testicular development in migrant and spawning bluefin tuna (*Thunnus thynnus* (L.)) from the eastern Atlantic and Mediterranean. Fish Bull 102 (407–417).

[130] Di Natale A (2011) ICCAT GBYP. Atlantic-wide Bluefin Tuna Research Programme 2010. GBYP Coordinator Detailed Activity Report for 2009–2010. Col. Vol Sci Pap ICCAT 66 (2): 995–1009.

