# Supplementary figures and images for: Electronic Tagging of Atlantic Bluefin Tuna (Thunnus thynnus, L.) Reveals Habitat Use and Behaviors in the Mediterranean Sea
Source: PLoS One. 2015 Feb 11;10(2):e0116638. doi: 10.1371/journal.pone.0116638 (PMC4324982; doi:10.1371/journal.pone.0116638)

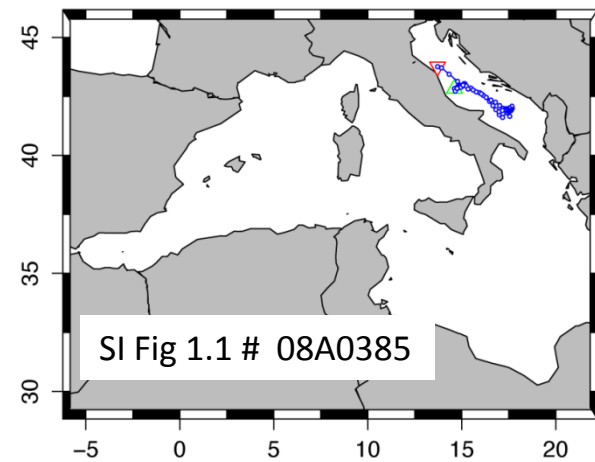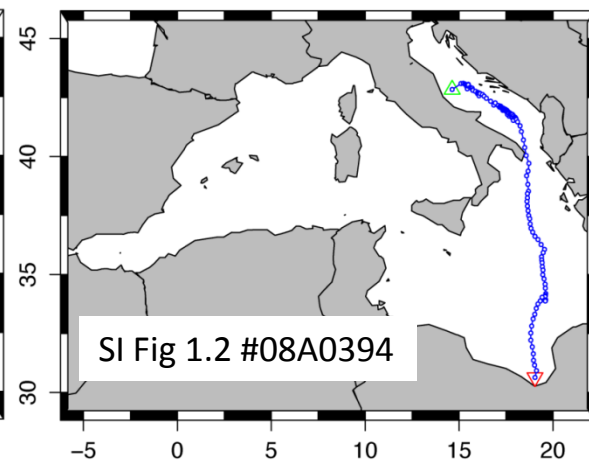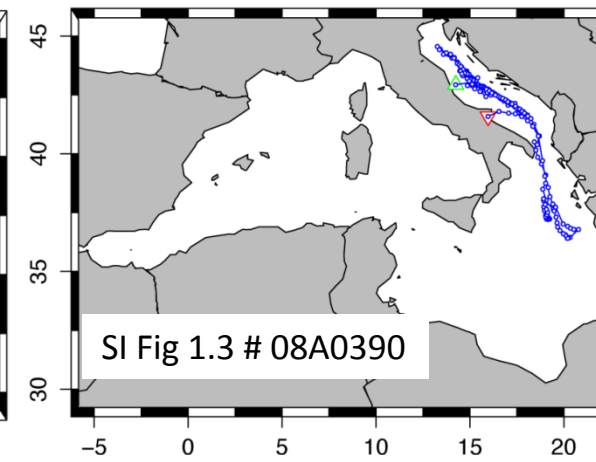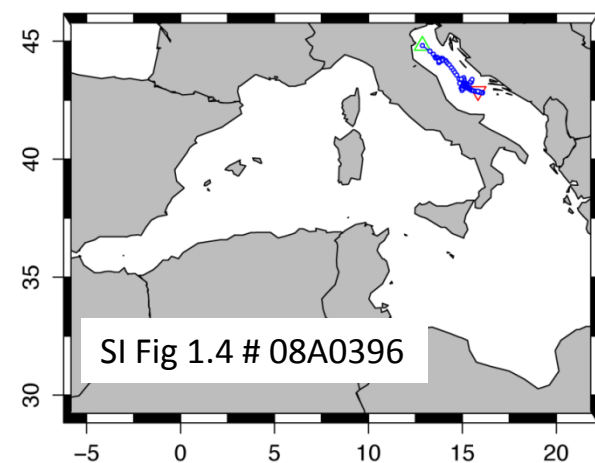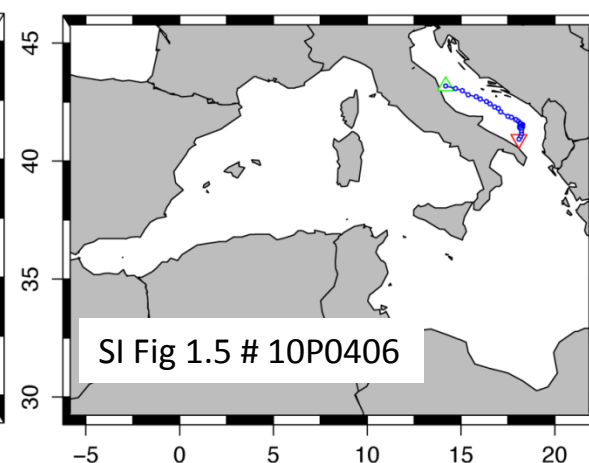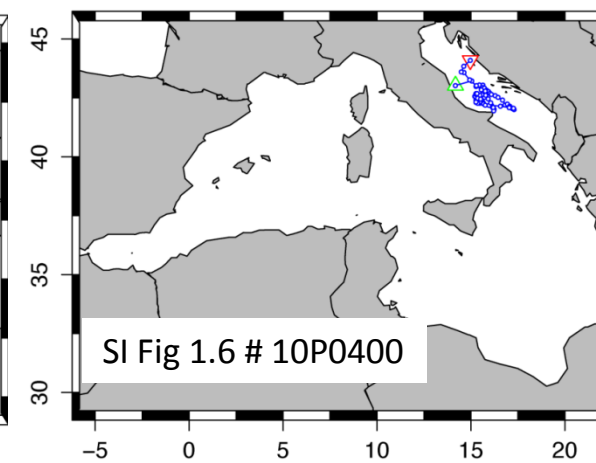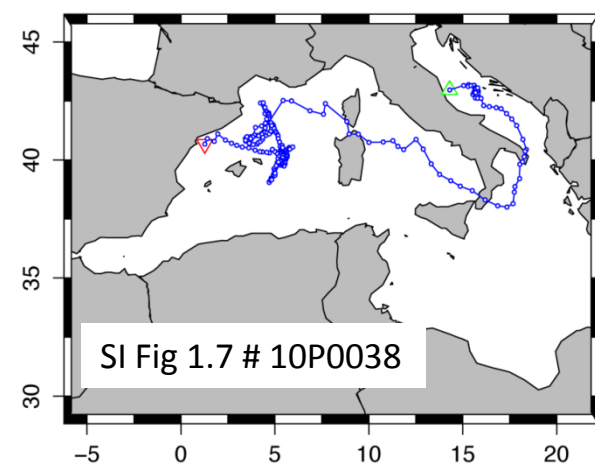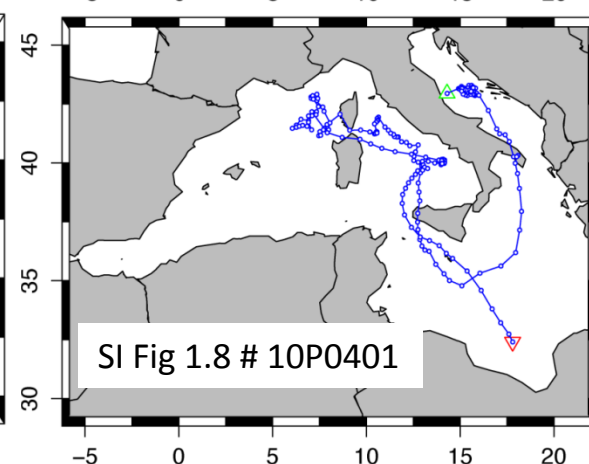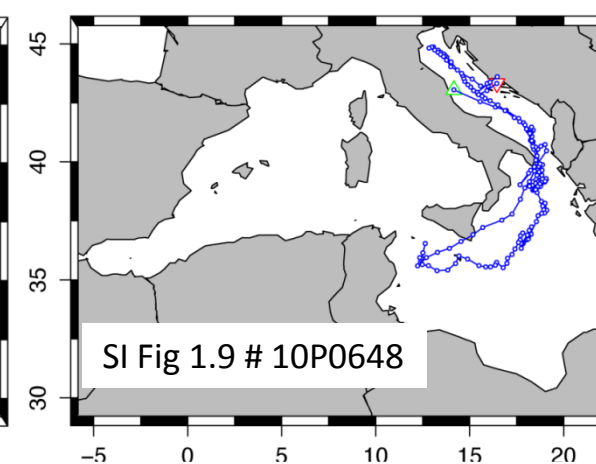

Supplement: S1 Fig — (PDF) [file pone.0116638.s001.pdf]

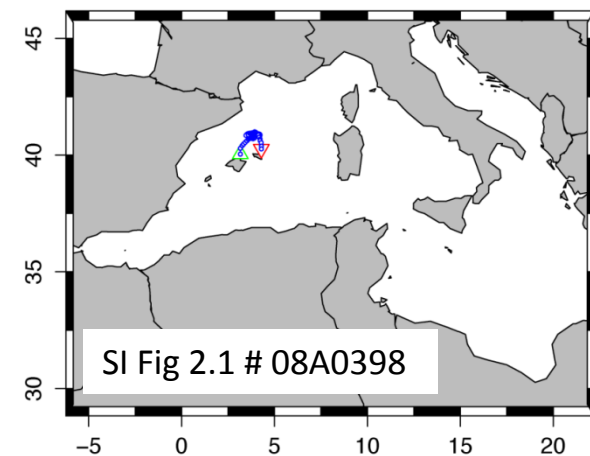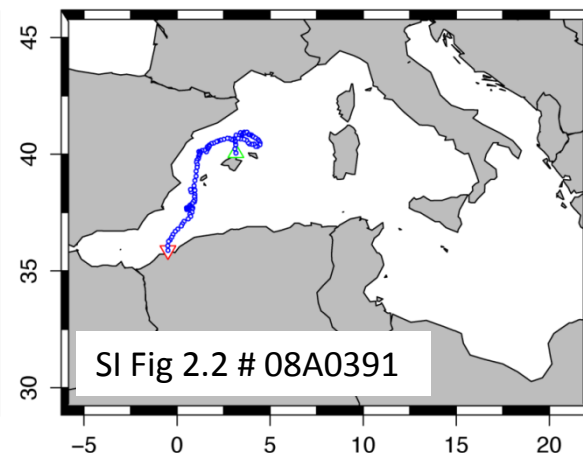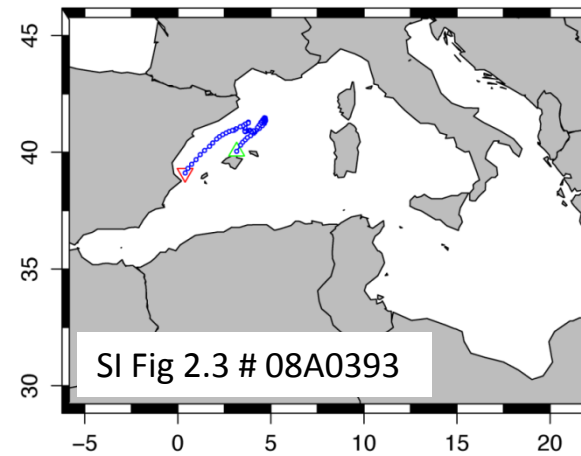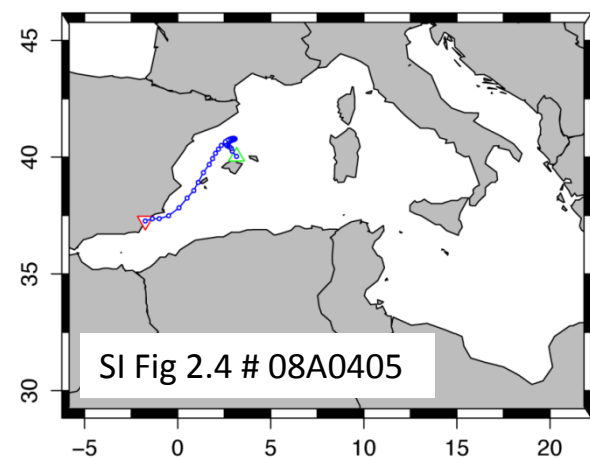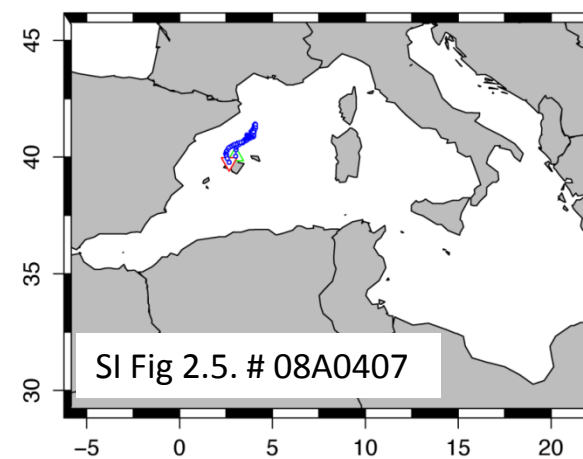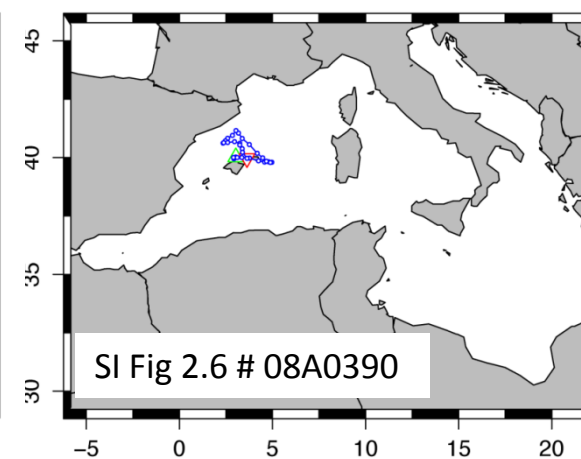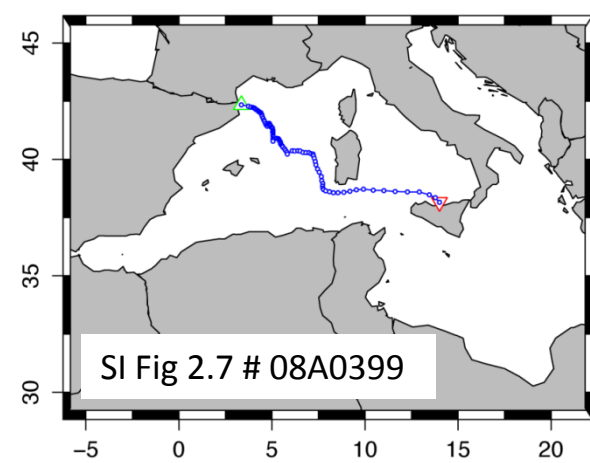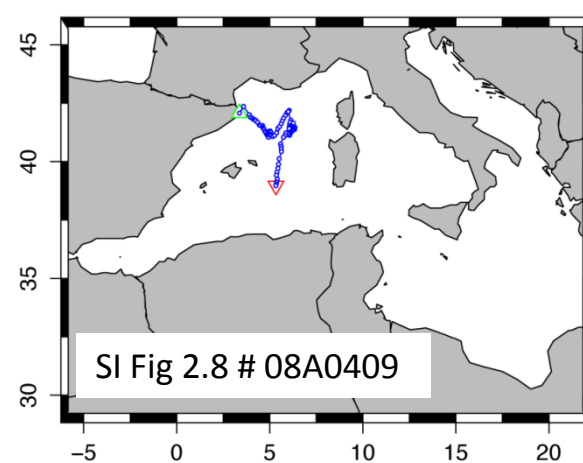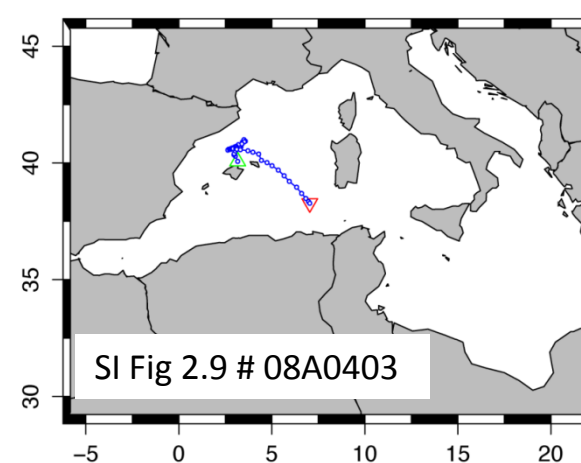

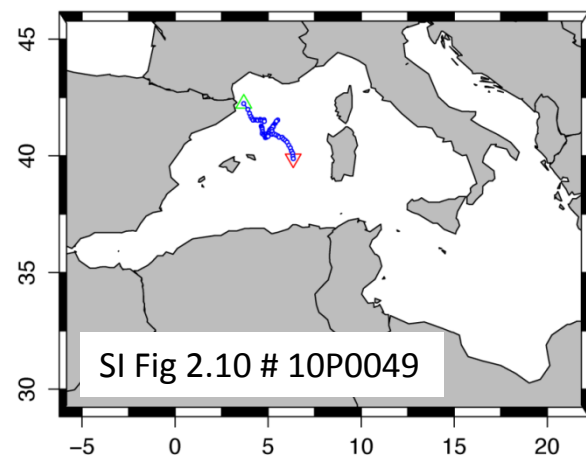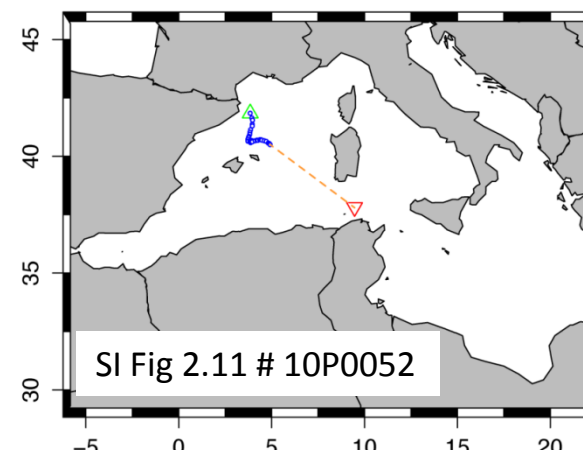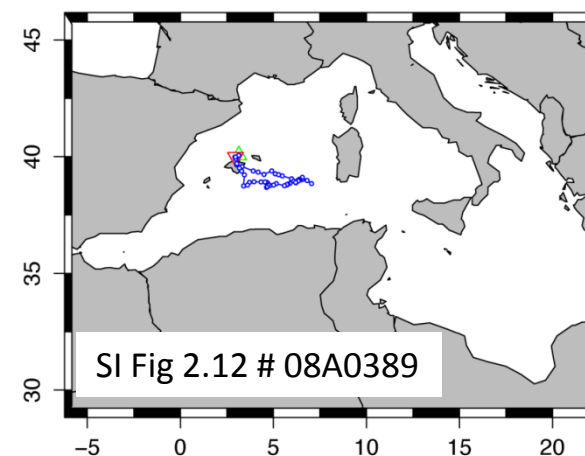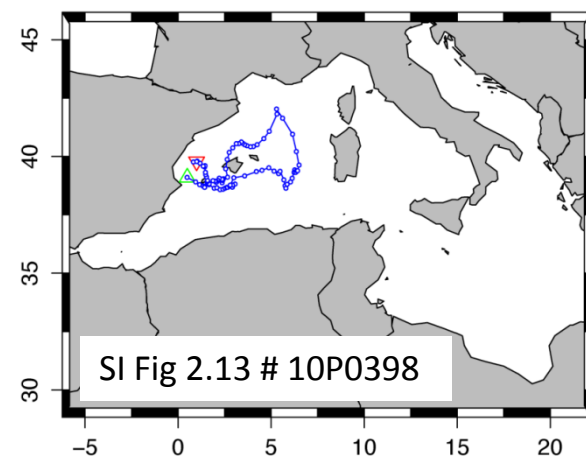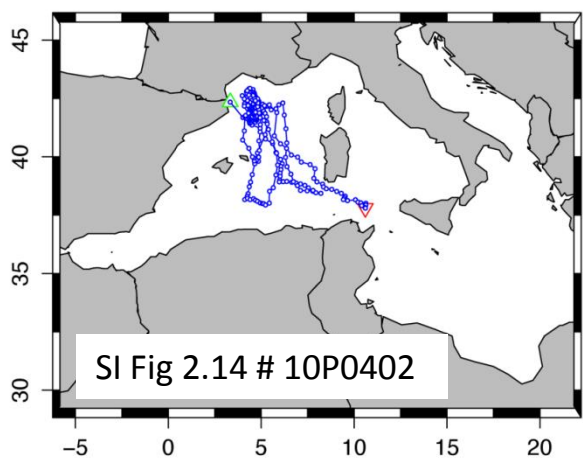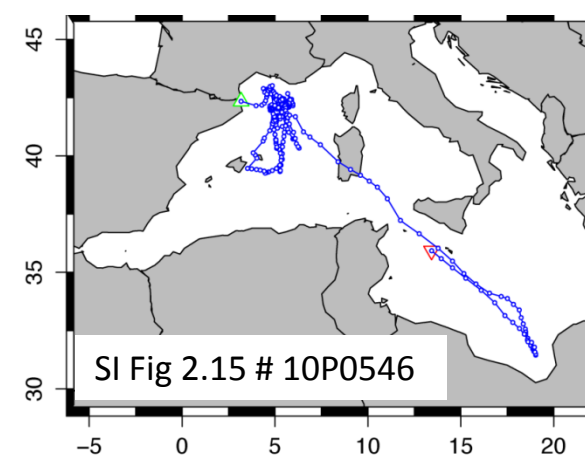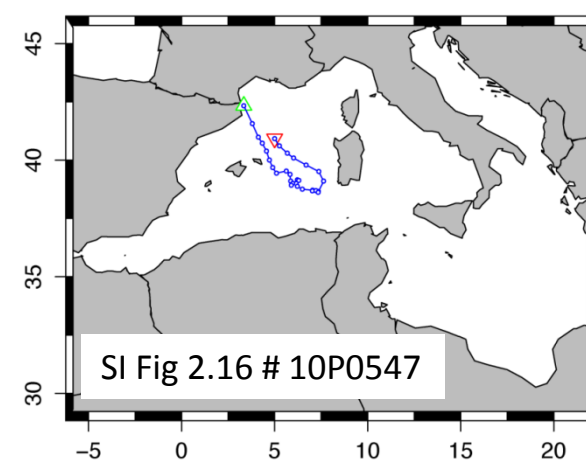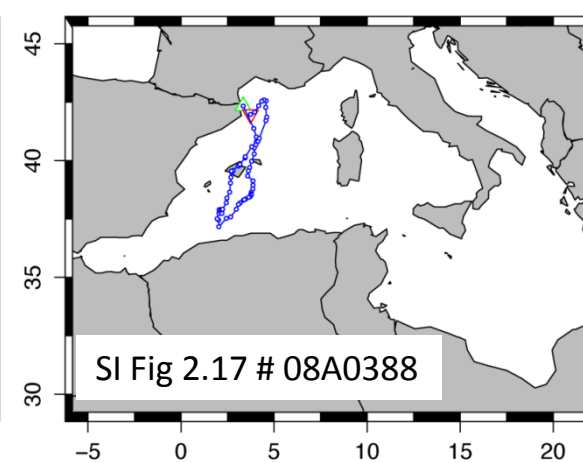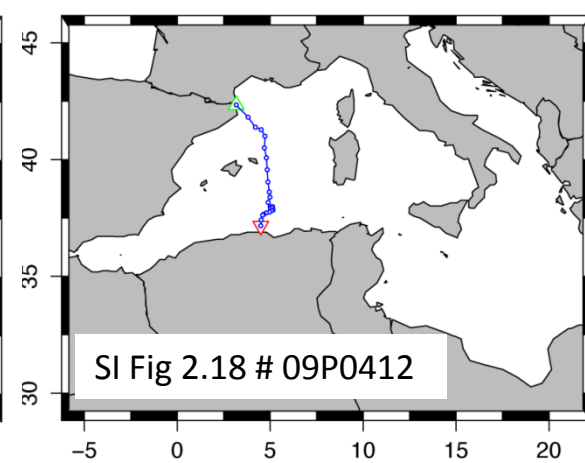

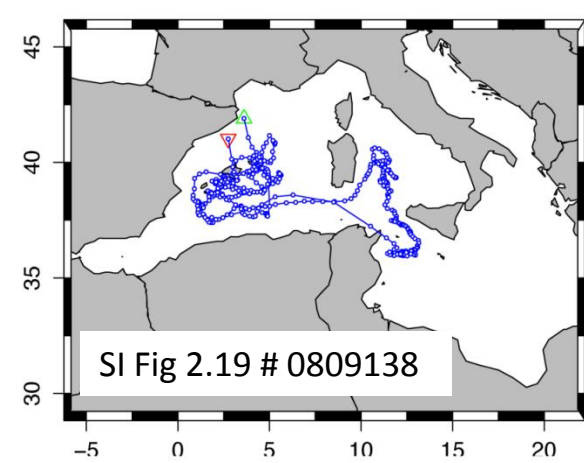

Supplement: S2 Fig — (PDF) [file pone.0116638.s002.pdf]

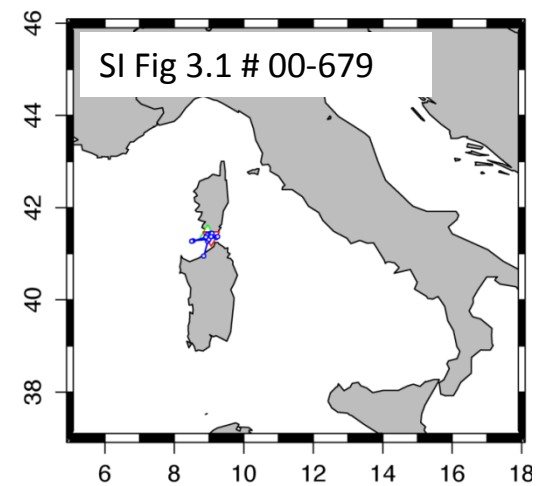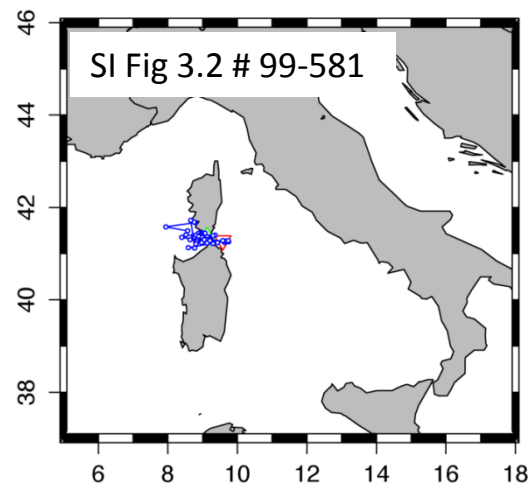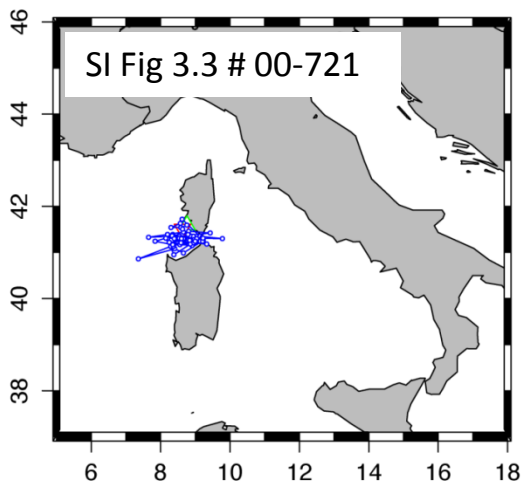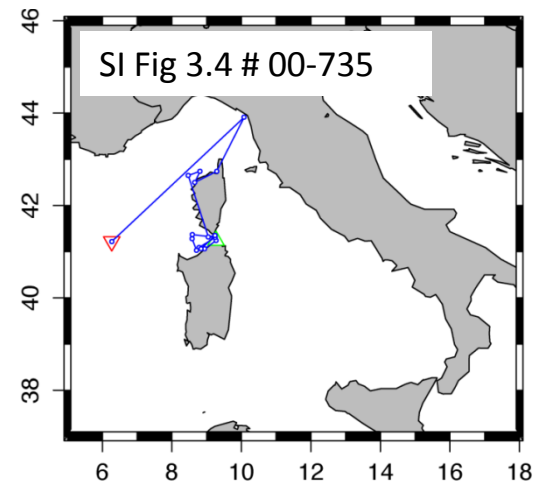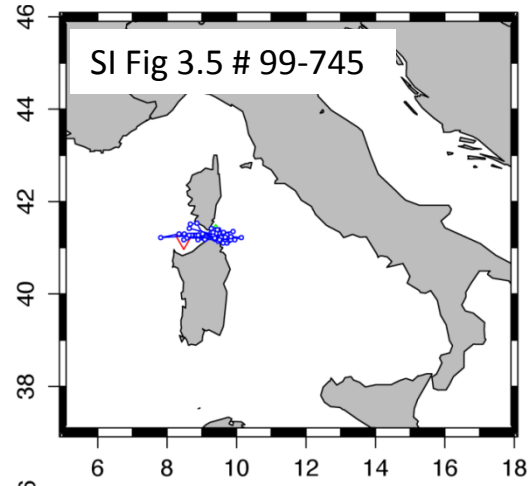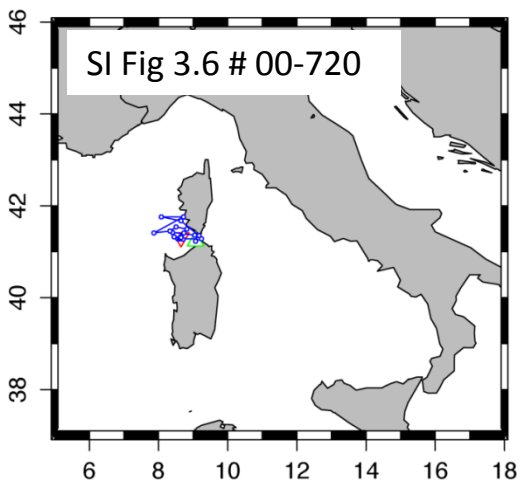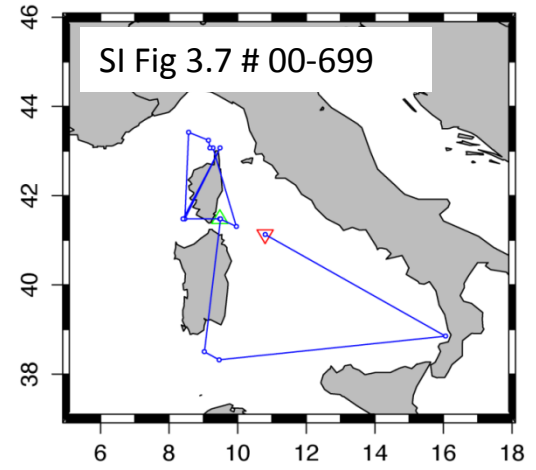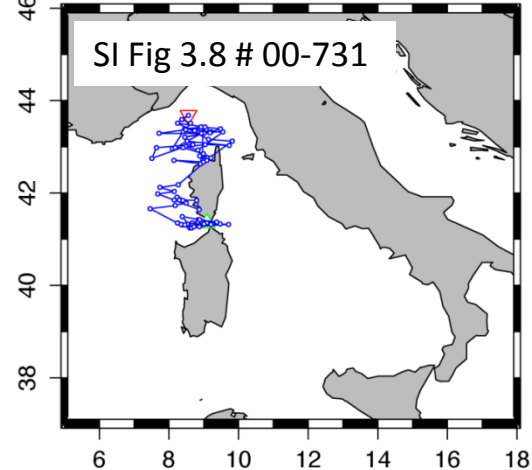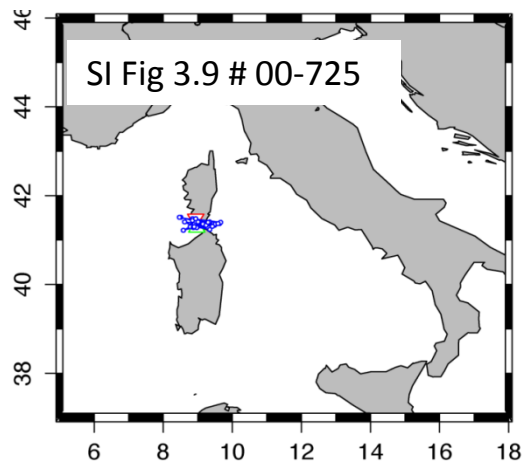

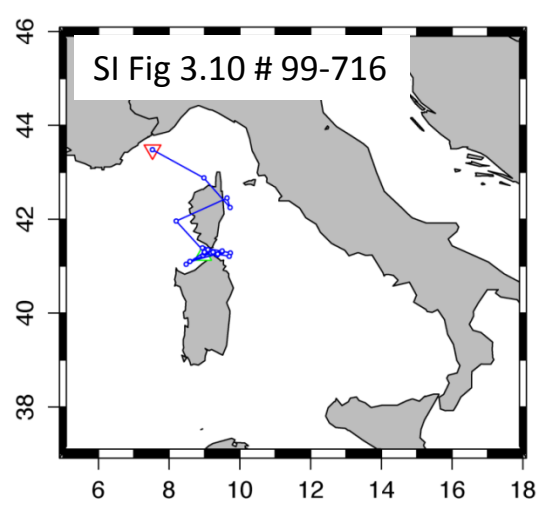

Supplement: S3 Fig — (PDF) [file pone.0116638.s003.pdf]
