# Supplementary material for: Electronic Tagging of Atlantic Bluefin Tuna (Thunnus thynnus, L.) Reveals Habitat Use and Behaviors in the Mediterranean Sea
Source: PLoS One. 2015 Feb 11;10(2):e0116638. doi: 10.1371/journal.pone.0116638 (PMC4324982; doi:10.1371/journal.pone.0116638)
Supplement: S1 Table — (DOCX) [file pone.0116638.s004.docx]

|  | **10P0398** | | **10P0546** | | **10P0402** | | **10P0648** | |
| --- | --- | --- | --- | --- | --- | --- | --- | --- |
|  | **Var (Lon)** | **Var (Lat)** | **Var (Lon)** | **Var (Lat)** | **Var (Lon)** | **Var (Lat)** | **Var (Lon)** | **Var (Lat)** |
| **Mean** | 0.64 | 0.34 | 0.76 | 0.32 | 0.64 | 0.28 | 0.35 | 0.28 |
| **SD** | 0.17 | 0.12 | 0.22 | 0.12 | 0.21 | 0.09 | 0.14 | 0.14 |
| **Min** | 0.00 | 0.00 | 0.00 | 0.00 | 0.00 | 0.00 | 0.00 | 0.00 |
| **Max** | 0.89 | 0.54 | 1.32 | 0.70 | 1.20 | 0.56 | 0.66 | 0.69 |
